# Molecular Insights into Cell-Mediated Immunity in Atypical Non-Ulcerated Cutaneous Leishmaniasis

**DOI:** 10.3390/microorganisms13020413

**Published:** 2025-02-13

**Authors:** Luís Fábio S. Batista, Carmen M. Sandoval Pacheco, Gabriela V. Araujo Flores, Frederico M. Ferreira, André N. A. Gonçalves, Wilfredo H. Sosa-Ochoa, Vânia L. R. da Matta, Claudia M. C. Gomes, Concepción Zúniga, Carlos E. P. Corbett, Daniel C. Jeffares, Helder I. Nakaya, Fernando T. Silveira, Márcia D. Laurenti

**Affiliations:** 1 Department of Pathology, Medical School, University of São Paulo, São Paulo 01246-903, Brazilgomescla@usp.br (C.M.C.G.); mdlauren@usp.br (M.D.L.); 2 Department of Clinical and Toxicological Analyses, University of São Paulo, São Paulo 05508-000, Brazil; 3 Microbiology Research Institute, National Autonomous University of Honduras, Tegucigalpa 11101, Honduras; wilfredo.sosa@unah.edu.hn; 4 Department of Health Surveillance, National Autonomous University of Honduras, Tegucigalpa 05005, Honduras; 5 Department of Biology, York Biomedical Research Institute, University of York, York YO31 5DD, UK; daniel.jeffares@york.ac.uk; 6 Hospital Israelita Albert Einstein, São Paulo 05620, Brazil; 7 Evandro Chagas Institute, Belém 66093-020, Brazil

**Keywords:** atypical non-ulcerated cutaneous leishmaniasis, visceral leishmaniasis, *L.* (*L.*) *infantum chagasi*, transcriptome, cell-mediated immune response

## Abstract

*Leishmania* (*Leishmania*) *infantum chagasi* infections range from asymptomatic (AS) to severe visceral leishmaniasis (VL). One of the manifestations is an atypical non-ulcerated cutaneous leishmaniasis (NUCL), which occurs in some locations of Central America with few cases of VL. We conducted a transcriptomic analysis of cell-mediated immunity (CMI) on blood samples from NUCL, AS, VL patients from Amapala, Honduras, and healthy controls. RNA-seq revealed a similar perturbation of gene expression in NUCL and AS. Eight gene signatures of CMI were found in NUCL involved in CD8^+^ T lymphocyte infiltration, reactive oxygen species generation, PD-1 receptor ligand, inflammasome assembly, chemotaxis, complement receptor and suppressor immune cell infiltration. NUCL was distinguished from VL by its up-regulation of differently expressed genes (DEGs) related to T lymphocyte exhaustion, adhesion and transmigration of leukocytes, and down-regulation of oxidative stress genes. In contrast, VL exhibited up-regulated DEGs involved in antigen cross-presentation, and similar to VL from Brazil, down-regulated DEGs involved in innate immunity. Corroborating the transcriptome findings, both the Leishmanin skin test, and the immunopathology of NUCL skin lesion defined NUCL as a proinflammatory condition, intermediate between the AS and VL clinical outcomes. That condition may be the underlying element for the benign nature of the NUCL.

## 1. Introduction

*Leishmania* (*Leishmania*) *infantum chagasi* infection presents a variety of clinical outcomes, typically ranging from asymptomatic infections to severe visceral leishmaniasis (VL) or atypical ulcerated cutaneous leishmaniasis (CL). However, in some countries of Central America, such as Costa Rica, El Salvador, Nicaragua, and Honduras, *L.* (*L.*) *infantum chagasi* predominantly causes non-ulcerated cutaneous leishmaniasis (NUCL) [1,2], and VL is rare. Between 2018 and 2020, 783 cases of NUCL and only 9 cases of VL were reported in Amapala, in the South of Honduras [3]. Although VL and NUCL are caused by *L.* (*L.*) *infantum chagasi*, they differ considerably in clinical presentation. VL is a chronic infection, with ineffective immune response and tissue damage in multiple organs [4,5], whereas NUCL is a localized, non-ulcerative cutaneous lesion, with a well-controlled immune response that sustains a low parasite burden [1,6]. Whereas VL can lead to death if neglected, NUCL can persist for several months or years without any previous history of VL or immunosuppressive disease [1,4]. NUCL is notable due to the non-stigmatizing nature of the lesions [1,4].

The incidence of atypical leishmaniasis has led to interest in its causative factors, as well as implications for treatment and control [7]. Clinical variation is related to several factors such as co-infections, treatments, and genetic variation of the parasite [8,9,10,11,12]. Immunosenescence, immunocompromise, general health, and concomitant diseases have been associated with aberrant presentations of cutaneous leishmaniasis [8,9,10]. Despite being classified as viscerotropic, cases of CL caused by species of the *Leishmania donovani* complex are observed in a variety of locations. For example, ulcerated CL is caused by *L.* (*L.*) *infantum chagasi* in countries in North Africa [11], ulcerated and non-ulcerated CL in Mediterranean basin [12], ulcerated CL in Brazil [13,14], and CL caused by *L. donovani* in India and Sri Lanka [15,16]. The Sri Lanka cases have been related to hybridization between *L. donovani* and dermotropic species such as *L. major* and *L. tropica* [16]. Unlike other atypical forms, NUCL represents a non-stigmatizing resistance to a species that usually causes severe VL within the same geographical region. This set of characteristics makes NUCL an interesting research object to improve the understanding of the parasite–host interaction. Such investigations may uncover the mechanisms underlying the local containment of the infection, the absence of ulceration, and the visceralization.

Transcriptomic analysis has been an essential tool in uncovering the molecular mechanisms of various forms of leishmaniasis [5,17,18]. Previous studies have provided insights into the gene expression profiles of VL in Brazil, identifying key pathways and genes such as those involved innate immune system associated with resistant profile and in antigen presentation, and chronic inflammation associated with susceptibility [5].

Previous studies have shown that NUCL lesions exhibit a predominantly pro-inflammatory response, with increased levels of Th1 and Th17 cytokines, alongside a regulatory balance maintained by Treg cells [3]. While the immune response in NUCL has been explored using techniques like ELISA, immunohistochemistry, and flow cytometry [6,19,20,21], there has been no transcriptomic analysis to date.

In this study, we conduct the first transcriptomic analysis of blood samples from NUCL patients, comparing them to asymptomatic *L.* (*L.*) *infantum chagasi* infection, VL patients from the same region, and healthy controls. Our findings revealed distinct differences in the cell-mediated immune response at the molecular level between NUCL and VL. These differences highlighted mechanisms such as leukocyte migration, T cell exhaustion, innate immunity, and the regulation of oxidative stress that likely contribute to the non-ulcerative, localized nature of NUCL compared to the more severe systemic involvement seen in VL. These insights contribute to a deeper understanding of host–parasite interaction during *L.* (*L.*) *infantum chagasi* infections.

## 2. Materials and Methods

### 2.1. Sampling

The sample collection was carried out in the Municipality of Amapala (13°15′37.1″ N 87°37′27.8″ W), Department of Valle, Gulf of Fonseca, South of Honduras. This is an endemic area of VL and NUCL caused by *L.* (*L.*) *infantum chagasi*. It is a region of dry tropical forest with mountainous and rugged topography, with an average altitude of 44 m. The temperature varies between 25 °C and 35 °C, with an average annual rainfall of 2096 mm. With the support of the Health Unit of the Municipality of Amapala, we selected for blood sample collection [10] (a) 10 patients presenting cutaneous injury of NUCL and positive immunological (LST and ELISA) and parasitological diagnosis by *L.* (*L.*) *infantum chagasi* HSP70 PCR-RFLP, (b) 9 asymptomatic individual with no clinical signs of NUCL nor VL, but with positive immunological and parasitological diagnosis of *L.* (*L.*) *infantum chagasi* infection (AS), and (c) 2 patients presenting symptoms of VL with positive parasitological and serological diagnosis from Amapala, free of specific treatment. Blood samples from 7 health donors from Municipality of São Paulo, Brazil, with a negative immunological and parasitological diagnosis for *Leishmania* infection were used as negative control (NEG). We chose São Paulo residents as infection negatives because the high frequency of serologically positive Amapala residents precluded confident selection of local negative controls. Patients were invited to participate in the study, and those who agreed were submitted to the collection of biological material after signing the consent form. The research project was approved by the Research Ethics Committee of the Faculty of Medicine of the University of São Paulo (CAEE protocol 55381216.0.0000.0065) and by the Research Ethics Committee of the National Autonomous University of Honduras (protocol 03-2014). After clinical and laboratory diagnosis NUCL, VL, AS, and NEG individuals selected were submitted to whole blood collection for transcriptome study.

### 2.2. Immunological and Molecular Diagnosis

Diagnosis was carried out using the Leishmanin Skin Test (LST), serology by ELISA for IgG and IgM detection using specific *L.* (*L.*) *infantum chagasi*-antigen, and *L.* (*L.*) *infantum chagasi* was identified in the culture of parasites isolated from the scraping of the cutaneous lesion (NUCL), and buffy coat (VL) by PCR-RFLPs through Hae III digestion of a Hsp70 fragment according to Sosa-Ochoa et al., 2020 [20].

### 2.3. Steps of RNA Sequencing, Differential Expression and Functional Enrichment Analysis

Blood samples were collected in PAXgene^®^ Blood RNA tubes (Qiagen, Germantown, MD, USA) and the total RNA was isolated using PAXgene blood RNA Kit (Qiagen GmbH, Hilden, NW, Germany), according to the manufacturer’s instructions. RNA quality and integrity were assessed by the Tape Station 4200 system (Agilent Technologies, Santa Clara, CA, USA). cDNA was prepared using TruSeq Stranded total RNA (Illumina, San Diego, CA, USA) with Ribo-Zero Globin magnetic beads (Illumina, USA) to deplete globin-encoding mRNA in addition to the cytoplasmic and mitochondrial rRNA. The RNA sequencing was carried out in the HiSeq 2500 Illumina platform. Sequenced reads were submitted to quality assurance with the FASTQC program version 0.11.9 [22]. Resulting sequences were mapped against the reference human genome (assembly GRCh38.p13 release 107) and counted with the program STAR version 2.7.1a [23]. Read counts were submitted to batch effect correction with the R package LIMMA version 3.60.0 [24], following quantile normalization and differential expression analysis with the R package EdgeR 4.2.0 [25]. Genes were considered differently expressed when adjusted *p*-value ≤ 0.05 (false discovery rate correction). Functional enrichment analyses were performed with the Bioconductor R-package FGSEA 1.30.0 [26] against the REACTOME version 2023 [27] and BTM version 2013 [28] databases of molecular pathways. The co-expression analysis was carried out with program CEMITOOL version 1.28.0 [29]. And the immunological cells types were deconvoluted with the program CIBERSORTX (https://cibersortx.stanford.edu accessed on August 2024) [30].

### 2.4. Comparison of Biomarkers of Immune Response Profiles and Lymphocyte Exhaustion

Lists of biomarkers were searched in databases such as InnateDB (https://www.innatedb.com/ accessed on October 2023), Mouse Genome Informatics (MGI) gene ontology list (https://www.informatics.jax.org/vocab/gene_ontology/GO:0045087 accessed on October 2023) and in the literature related to immune response profiles Innate, Th1, Th17, Th2, CTL, Treg immune response and T lymphocyte exhaustion (https://pubmed.ncbi.nlm.nih.gov/ accessed on October 2023). The log-value of expression of each biomarker were compared between NUCL and VL (a), NUCL and NEG (b), and VL and NEG (c) by Student’s *t* test in the case of a normal distribution of data or Mann–Whitney test in the case of a non-normal distribution of data on GraphPad Prism v8.0.1.

### 2.5. Immunohistochemistry Study

Immunohistochemistry was performed on the 23 skin biopsies of patients affected by NUCL and 10 normal skin biopsies from healthy individuals recruited and diagnosed as described in Sandoval Pacheco et al., 2023 [3]. The cellular density between NUCL and NEG were compared by Student’s test *t* or Mann–Whitney on GraphPad Prism v8.0.1.3.

## 3. Results

**Figure 1:**
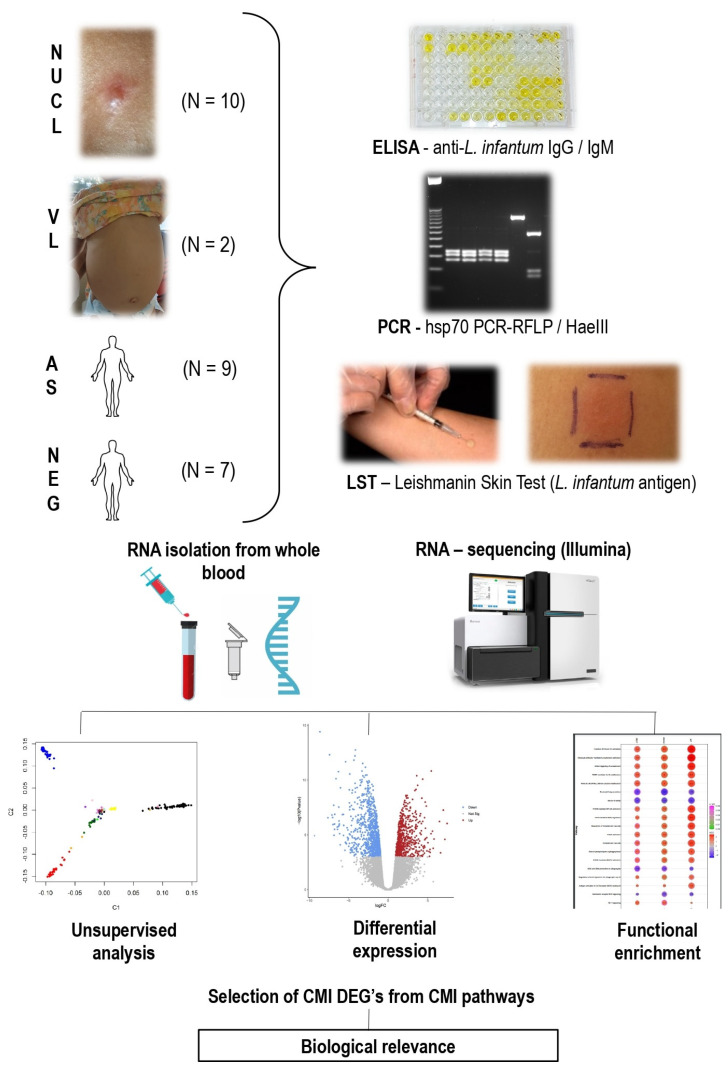
Diagram summarizing the sampling, diagnostic methods (ELISA—Enzyme Linked ImmunoSorbent Assay, PCR—Polymerase Chain Reaction, LST—Leishmanin Skin Test) and transcriptome analysis by RNA-sequencing of non-ulcerated cutaneous leishmaniasis (NUCL), visceral leishmaniasis (VL) patients, asymptomatic (AS) from Amapala, Honduras and non-infected (NEG) individuals from São Paulo, Brazil. Identification of cell-mediated immunity (CMI) differentially expressed genes (DEGs) associated with different clinical outcomes. N: Number of cases studied per group.

In previous studies, our group demonstrated the clinical and immunopathological features of the natural infection by *L.* (*L.*) *infantum chagasi* in human patients from Amapala, Honduras [6,19,20,21]. To better understand the molecular mechanisms of cell-mediated immunity (CMI) behind the mild clinical outcome that does not ulcerate and does not visceralize in NUCL, we performed transcriptome RNA-seq for blood samples of 10 patients with non-ulcerative cutaneous leishmaniasis (NUCL), 2 patients with visceral leishmaniasis (VL), and 9 asymptomatic patients (AS) who were recruited, examined and diagnosed in Amapala—Honduras [20], and 7 uninfected negative controls (NEG) from São Paulo—Brazil. The limited number of samples from VL patients is because VL is very rare in Amapala [3]. Table S1 presents patients data. Figure 1 summarizes the methodology.

### 3.1. RNAseq Analysis and Number of Differentially Expressed Genes (DEGs)

**Figure 2:**
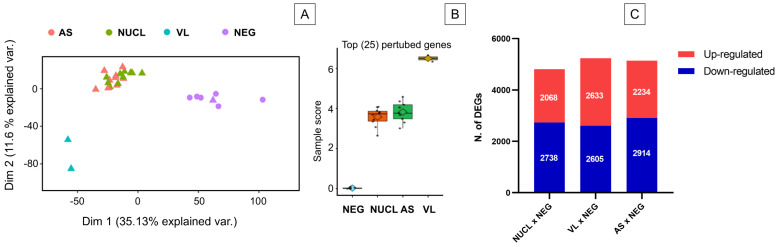
Unsupervised analysis and number of differentially expressed genes (DEGs) for the RNA-seq of whole blood samples from non-ulcerated cutaneous leishmaniasis (NUCL), visceral leishmaniasis (VL), asymptomatic (AS), non-infected (NEG) individuals. (**A**) Principal Component Analysis of expression level show 3 defined clusters. NULC samples clustered along Asymptomatic individuals in a distinct cluster from NEG and VL. Triangle: individual from Honduras. Circle: individual from Brazil. (**B**) Comparison of the top 25% of perturbed genes among the groups NEG, AS, NUCL, and VL show that changes in gene expression in NUCL is similar to AS and distinct of NEG and VL. (**C**) Bar plot presents the numbers of up and down-regulated differentially expressed genes (DEGs) in the groups NUCL, VL and NEG when compared to the NEG group. Differential expression was defined as 2-fold change Log of expression. Up-regulated genes are the genes with the most expression in the infected groups and down-regulated genes are the genes most expressed in the negative group.

To characterize differences between leishmania infection manifestations and controls, an unsupervised principal component analysis (PCA) was performed using the global expression data of all genes from the RNAseq. The PCA revealed three clusters: a first cluster including NUCL and AS, a second of NEG, and a third one of VL individuals (Figure 2A). Importantly, NUCL patient samples clustered closely with asymptomatic (AS) *L.* (*L.*) *infantum chagasi* infections. To determine the molecular perturbation caused by the *L.* (*L.*) *infantum chagasi* infection in NUCL, AS, and VL cases relative to NEG cases, we submitted the normalized gene expression to the Molecular Degree of Perturbation (MDP) webtool [31]. The degree of perturbation of the top 25% perturbed genes was comparable in NUCL and AS and but far higher in VL individuals (Figure 2B). Hence, both PCA and MDP showed that overall gene expression between the groups NUCL and AS were similar. To determine which genes had altered expression in AS, NUCL and VL cases, we identified the differentially expressed genes for each infected group relative to the negative controls (NEG), using a threshold of 2-fold change for differently expressed genes (DEGs), with an adjusted *p*-value < 0.05 (FDR) from Benjamini–Hochberg correction. This included 4806 DEGs between NUCL and NEG (2068 up-regulated and 2738 down-regulated), 5238 between VL and NEG (2633 up-regulated and 2605 down-regulated), and 5148 between AS and NEG (2234 up-regulated and 2914 down-regulated) (Figure 2C). The numbers of differentially expressed genes were similar among the comparisons (Figure 2C).

### 3.2. Identifying Gene Signature of Cell-Mediated Immunity in NUCL

**Figure 3:**
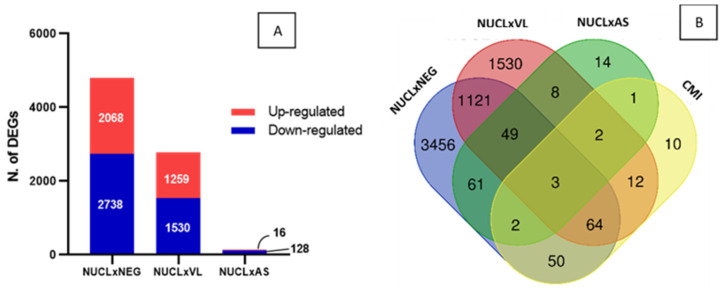
Identification of gene signature of cell-mediated immunity in NUCL cases by counting CMI DEGs in the NUCL vs. AS. (**A**) The number of DEGs counting for the datasets NUCL vs. NEG, NUCL vs. VL, NUCL vs. AS confirms the wide similarity between the groups NUCL and AS. (**B**) Venn diagram shows the identification of 8 CMI DEGs of NUCL vs. AS (green balloon), 2 in intersection with NUCL vs. NEG, 3 in intersection with NUCL vs. NEG and NUCL vs. VL, 2 in intersection with NUCL vs. VL, and only one DEG exclusive of NUCL vs. AS. This 8 DEGs were identified as transcriptomic signature of cell-mediated immunity in NUCL (Table 1).

The identification of gene signatures of cell-mediated immunity (CMI) exclusive to NUCL could be difficult in a scenario of high number of DEGs in the three datasets tested (Figure 2C). Therefore, we proceeded to a method that takes advantage of the equivalent gene expression perturbation caused by *L.* (*L.*) *infantum chagasi* infection in both NUCL and AS (Figure 2A,B). We adopted the following hypothesis: if the expression of a set of genes differs between clinical forms with such similar expression profiles, the DEGs would consistently characterize these clinical forms. To confirm the similarity between NUCL and AS, we compared the number of DEGs between NUCL vs. NEG, NUCL vs. VL, and NUCL vs. AS (Figure 3A). The number of DEGs of the NUCL vs. NEG was greater than the number of DEGs from the NUCL vs. VL. The number of DEGs from NUCL vs. AS was the lowest, confirming the similarity (Figure 3A). From 140 DEGs that differed between NUCL and AS, apparently only 8 have direct involvement with CMI and were defined as CMI gene signatures of NUCL (Figure 3B—green balloon). Two of these DEGs were in intersection with NUCL vs. NEG, three in intersection with NUCL vs. NEG and NUCL vs. VL, two in intersection with NUCL vs. VL, and only one DEG exclusive of NUCL vs. AS (Figure 3B—green balloon). The CMI gene signatures in NUCL include *BATF2*, *SH3PXD2B*, *CD274*, *GBP1*, *CCR3*, *CR1*, *MARCO*, and *COLEC12*. All fold-change values (LogFC) of the expression for the NUCL group in relation to the groups AS, VL, NEG, and their functions are presented in Table 1.

Among the CMI gene signatures in NUCL, *CCR3* and *CR1*, whose LogFC of NUCL in relation to the VL was greater than the LogFC of NUCL in relation to the AS, were highlighted as protective genes since NUCL is thought as an intermediate resistance between AS and VL clinical outcomes. However, other genes have contrasting expression in NUCL (*SH3PXD2B* and *GBP*)., i.e., they were up-regulated in NUCL in comparison to the AS and down-regulated in NUCL in comparison to the VL. This finding indicates that a stronger regulation of these genes is required to avoid exacerbated inflammation, oxidative stress, and evolution towards the susceptible pole (VL).

### 3.3. Pathways Enrichment Analysis

Genes expressed in NUCL and VL were grouped in the functional enrichment analysis using the Reactome Immune System (RIS) and Blood Transcription Modules (BTM) databases to determine DEGs that helped identify differences in profile of gene expression between the datasets NUCL vs. NEG and VL vs. NEG. To perform a more pragmatic transcriptomic analysis focused on clinical differences between NUCL and VL, the group AS was removed due to the uncertainty of the clinical outcome of the group AS and the similarity between NUCL and AS (Figure 2).

Of 200 enriched pathways for the dataset NUCL vs. NEG, 41 presented statistically significant normalized enrichment scores (NES) (*p* < 0.05). From 41 significant pathways, 25 were clustered in the RIS (Figure S1A), and 16 were clustered in the BTM database (Figure S1B). From 198 pathways enriched for the dataset VL vs. NEG, 81 were statistically significant (*p* < 0.05), 36 were clustered in the RIS database (Figure S1C), and 45 were clustered in the BTM database (Figure S1D). Based on the normalized enrichment score (NES), 26 pathways were up-regulated (NES > 0), 15 were down-regulated (NES < 0) in NUCL vs. NEG dataset, 53 pathways were up-regulated (NES > 0), and 28 were down-regulated (NES < 0) in VL vs. NEG. Only DEGs belonging to pathways potentially related to the CMI were selected for functional description. From the NUCL vs. NEG dataset, we evaluated the functional annotation of 1376 genes related to CMI, 678 in BTM and 685 in RIS databases. Of the VL vs. NEG dataset, we evaluated the functional annotation of 1569 genes related to CMI, 744 in BTM and 825 in RIS databases.

### 3.4. Comparison of DEGs for CMI Between NUCL and VL

**Figure 4:**
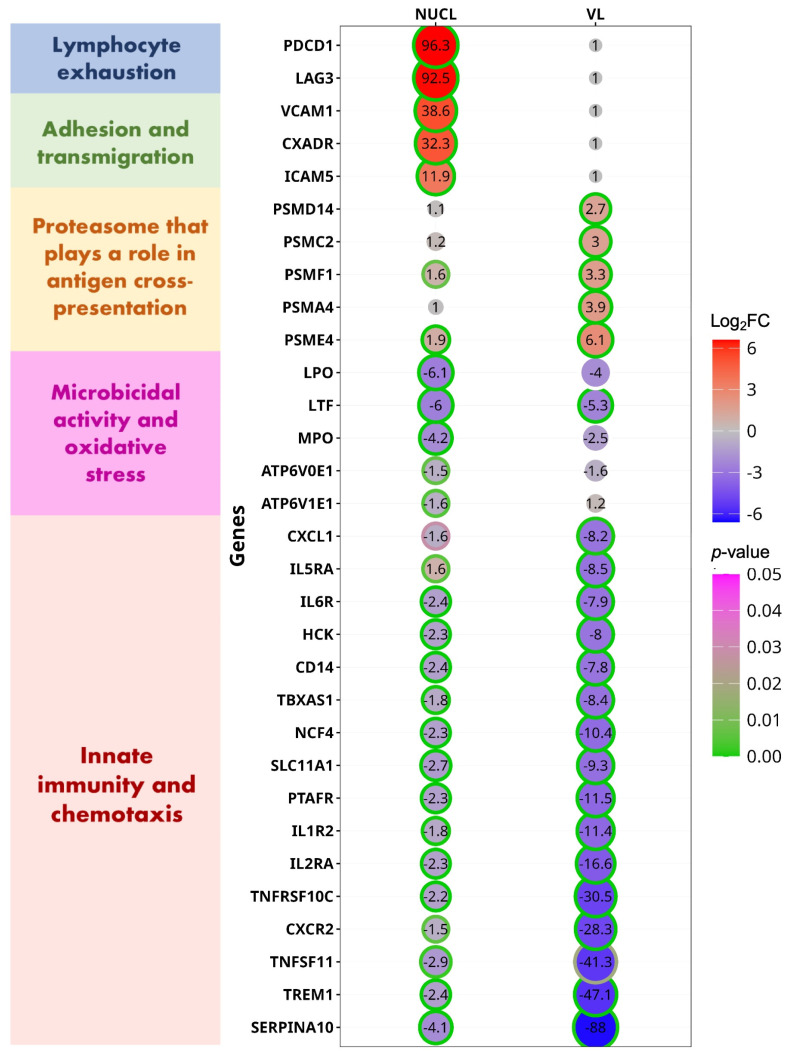
Circle plot demonstrates the differently expressed genes (DEGs) related to cell-mediated immunity (CMI) for the datasets NUCL vs. NEG and VL vs. NEG. DEGs are presented in groups by the role played in the CMI. Each DEG was selected from the enriched pathways functionally related to CMI for the datasets NUCL vs. NEG and VL vs. NEG. The Log fold-changes (logFCs) of each DEG are presented inside of each circle and is represented by the size of each circle. The ring edge of each circle represents the statistical significance (*p*-value) of the LogFC of each DEG related to the NEG group.

It is possible that the VL caused by *L.* (*L.*) *infantum chagasi* strains from Honduras is atypical of VL caused by *L.* (*L.*) *infantum chagasi* from other locations in South America. To examine this, we compared the gene expression changes from the two VL cases from Honduras (vs. NEG) in the present study to gene expression changes from an RNAseq data set of 10 VL cases from Brazil [5]. We found that 83/126 (66%) of the DEGs involved in CMI from Honduras were also differentially expressed in the VL cases from Brazil [5], 79/83 (95%) of these DEGs were up/downregulated in the same direction between Honduras and Brazil cases. This analysis reinforces the consistency of the statistical analysis in the present study and indicates that VL from Honduras is very similar to VL from Brazil. In contrast, NUCL patients differed considerably from VL in upregulated CMI genes, consistent with the PCA analysis. From the 63 CMI genes that were upregulated in either NUCL or VL, 19 were upregulated exclusively in NUCL patients, 16 were exclusively in VL patients, and 28 genes were DEGs in both NUCL and VL (Figure 4, Table S2).

Among the selected up-regulated genes exclusive to NUCL, the two with highest expression changes were the Programmed Cell Death 1 (*PDCD1*, logFC = 6.58) and Lymphocyte Activating 3 (*LAG3*, logFC = 6.53). *PDCD1* (synonym PD1) encodes an inhibitory surface receptor critical for the regulation of activated T lymphocytes during immunity and tolerance. PD1 triggers inhibitory signals upon binding to ligands CD274/PD1L1 and CD273/PD1LG2 [32]. *LAG3* encodes an inhibitory receptor on antigen activated T-cells, which triggers inhibitory signals upon binding to FGL1 or acting as a coreceptor for PD1 [33].

T-cell exhaustion via inhibitory receptors expression during human and animal visceral leishmaniasis has been widely reported as a mechanism of persistent chronic infection and drug-resistance [18,34]. The blood expression of the T-cell exhaustion biomarkers such as *PDCD1*, *PDCD1LG2*, and *LAG3* confirmed the role of lymphocyte exhaustion in NUCL and in VL cases. From 50 T-cell exhaustion biomarkers evaluated, the expression of 20 biomarkers from the comparison VL vs. NEG and 14 from the comparison NUCL vs. NEG was significantly different (Figure S3, Table S2). This analysis of biomarkers indicates that T lymphocyte exhaustion occurs in both NUCL and VL via a simultaneous increase in expression of *PDCD1*, *PDCD1LG2*, *LAG3*, *CD86*, *STAT1*, and *TNF*. Conversely, increased expression of *IL10* occurred only in VL.

The third most up-regulated (logFC = 5.33) NUCL-exclusive DEG was CD1A (Table S3). The *CD1A* protein binds self and non-self-lipid or glycolipid antigens and presents them to the antigen receptor on lymphocytes and NK cells. Dendritic cells can initiate antimicrobial responses by CD1-mediated presentation of pathogen-derived glycolipids [35]. It has been attributed to *Leishmania* species the inhibition of CD1 expression [36,37]. VCAM-1 (vascular cell adhesion molecule-1) is a cytokine-inducible adhesion molecule that is known to mediate adhesion of mononuclear cells to endothelial cells in vitro via binding to the integrin VLA-4 (very late antigen-4) [38]. *CXADR* (logFC = 5.01) is involved in transepithelial migration of leukocytes [39]. ICAM5 (logFC = 3.57) proteins are ligands for the leukocyte adhesion protein LFA-1 [40].

COLEC12 (logFC = 3.37) is a scavenger receptor that promotes the binding and phagocytosis of bacteria and yeast [41]. It also correlated to infiltration of several immune cells, such as M2 macrophages, dendritic cells, neutrophils, and regulatory T cells, suggesting that it may also play a role in suppressing tumor immune response [42]. The most up-regulated DEGs to VL (*PSMD14*, *PSMC2*, *PSMF1*, *PSMA4*, *PSME4* and others (Figure 4, Table S3). That genes are part of a proteasome activation complex that make up the cross-presentation pathway of exogenous antigens on major histocompatibility complex (MHC) class I. MHC class I presentation of exogenous antigens is the mechanism enabling professional antigen-presenting cells (APCs) to induce CD8^+^ T-cell and natural killer (NK) cell responses against viruses and tumors that do not have access to the classical MHC class I pathway [43]. The CD8^+^ T cell response has been reported as an important source of cytokines in the immune response against visceral leishmaniasis as well as being marked by inhibition and exhaustion signatures in symptomatic VL [44]. This finding agrees with the up-regulation of the *CD8A* and *CD8B* genes in the VL and NUCL blood samples (Table S3) and increased count of IFN-γ-producing CD8^+^ and CD8^+^ cells in the NUCL cutaneous lesion [3].

The DEGs that were up-regulated in both VL and NUCL vs. NEG (Table S3) include genes involved in the activation, differentiation, and cytotoxicity of several T-cell subsets (*CDCA7*, *CRTAM*, *NCR1*); T cell receptor subunit (CD3D); CD8^+^ T lymphocyte co-receptors (CD8A and CD8B); MHC class II subunits (*HLA-DPA1*; *HLA-DRA*, *HLA-DQB2*, *HLA-DQB1*), and T cell inhibitory genes *(PDCD1LG2*, *KLRD1*, *KLRC1*, *CD274*). The T cell inhibitory genes were more expressed in NUCL than in VL.

From the 85 down-regulated CMI DEGs, 3 were exclusively expressed in NUCL patients, 21 were exclusively expressed in VL patients, and 61 genes were expressed in both NUCL and VL (Figure 4, Table S4). The CMI down-regulated DEGs exclusive of NUCL are involved with generation of reactive species and acidification of intracellular compartments, and are therefore implicated with microbicidal activities (*LPO*, *MPO*, *ATP6V0E1*) [45,46,47]. These findings of the down-regulation of genes involved in oxidative stress and microbicidal activities are consistent with a chronically persistent infection and the prevention of tissue damage caused by oxidative stress in the NUCL skin lesions, which are characterized by the absence of ulceration. The 21 down-regulated DEGs exclusive of VL patients enriched pathways such as IL-10 signaling, IL-3, IL-5, and GM-CSF signaling, which were down-regulated (NES < 0) in the functional enrichment analysis. This gene expression profile in blood samples corroborates the model of lower regulation of the immune response in clinical VL, which is characterized by exacerbated inflammatory responses and tissue damage in multiple organs. However, the functional enrichment for genes downregulated in VL was only impacted by other genes of the IL-10 pathway, since the gene for IL-10 itself was upregulated in VL patients (Figure S2D).

The most discrepant down-regulated DEGs between NUCL and VL cases (Figure 4, Table S4) include innate immunity genes (*SERPINA10*, *TREM1*, *CXCR2*, *CSF3R*, *TNFRSF10C*). These were more intensely suppressed in VL than in NUCL patients. SERPINA10 encodes a protease inhibitor correlated with leukocyte infiltration and immunological checkpoints [programmed cell death-1 (PD-1) and CTLA-4] in peripheral tissues [48,49]. *TREM1* encodes a receptor that plays an important pro-inflammatory role in acute and chronic inflammatory disorders, which is involved with myeloid cells infiltration [50]. *TNFRSF10C* or *TRAILR3* encodes the receptor for the apoptosis and pro-inflammatory response-inducing factor TRAIL, which has been associated with ulceration in cutaneous leishmaniasis [51].

### 3.5. Blood Gene Expression Shows a Stronger Pro-Inflammatory Response and Regulation in NUCL than in VL

The immune response in cases of NUCL has been described in depth in studies that evaluated the skin lesion. Knowledge about the immune response in the bloodstream remains scarce. To further understand the role played by the immune system in NUCL we compared expression of immune response biomarkers between NUCL and VL.

Because we do not know whether whole blood is suitable to evaluate the NUCL CMI transcriptome, we decided to test the blood expression of each Th1, Th17, T regulatory (Treg), Th2 and Cytotoxic CD8^+^ T Lymphocyte (CTL) immune response subsets of biomarkers to investigate if the immune response profile observed in the NUCL skin lesion is reflected in the in the blood stream.

For Th1 immune response (Figure S2A), significant differences were found in 20/51 biomarkers, of which *IFNGR1*, *IFNGR2*, *IFNG-AS1*, *IL23R*, *IL3RA*, *CXCR4*, *TRAF6*, *CSF2RA*, *CSF2RB*, *TNFRSF9*, *TNFRSF8*, *TNFRSF1B*, *TNFRSF10B*, *TNFRSF10C*, *TNFRSF1A*, *TNFRSF11A*, and *TNFRSF12A* were higher in NUCL than in VL (NUCL > VL). In contrast, only *IL12R1*, *STAT1*, and *CCR2* had higher expression in VL than in NUCL (VL > NUCL). This suggest that the immune response may be more proinflammatory in NUCL than in VL.

We found that 16/26 Th17 biomarkers were statistically different between NUCL and VL patients. These differences corroborate the more proinflammatory profile for NUCL (Figure S2B). From the 16 Th17 DEGs, 10 genes (*TGFB1*, *TGFB2*, *TGFBR1*, *TGFBR2*, *TGFBR3*, *IL6R*, *IL23R*, *IL17RA*, *STAT3*, *RORC*) had a higher expression in NUCL (NUCL > VL), whereas *IL6ST*, *IL23A*, *IL17D*, *IL17RB*, *SLAMF6*, and *CCR6* had a higher expression in VL (VL > NUCL). The increased expression of *IL6ST* and *IL17D* in VL patients is consistent with higher levels of the cytokines IL-6 and IL-17 released in the serum of VL compared to the NUCL patients [20]. This supports our expectation that RNAseq analysis from the blood samples of NUCL patients can describe relevant factors of the NUCL immune response.

Among the 28 biomarkers of CTL immune response (Figure S2C), only *GZMA* showed statistical difference between NUCL and VL, with higher expression in VL than in NUCL patients. Other CTL biomarkers with a statistical difference between VL and NUCL were shared biomarkers between CTL and Th1 immune responses. These data suggest a significant helper role of CD8^+^ T lymphocyte in NUCL.

For Treg immune response, 13/30 biomarkers showed significant difference between NUCL and VL (Figure S2D). Only IL10 was overexpressed (Figure S2D) in the blood and more secreted in the serum [20] of VL compared to NUCL individuals (VL > NUCL). Other biomarkers, such as the IL10 receptor, *TGFB*, *STAT5*, *BCL6*, *CD46*, and *KAT5*, were more highly expressed in NUCL (NUCL > VL). It is also consistent with a stronger control of proinflammatory response in NUCL.

The study of the Th2 immune response showed that 24/32 markers were significantly different between NUCL and VL (Figure S2E). Only *IL10*, *LTBP1*, and *DENND1B* showed higher expression in VL (VL > NUCL). Other genes related to IL-10 receptor, *IL-4*, *IL-5*, *IL-13*, *TGF-β*, *STAT6*, *CXCR4*, *CCR3*, *CCR4*, *CCR8*, *DIDO1*, *NLRP3*, and *NOD2* were higher in NUCL (NUCL > VL), reinforcing the idea of the stronger regulation of proinflammatory response in NUCL. In addition, the data strongly suggested that high levels of IL-10 contribute to the ineffectiveness of the immune response and greater clinical severity in VL.

Comparatively, the greatest differences between NUCL and VL were observed in the expression of innate immunity biomarkers (49 for innate immunity, 24 for Th2, 20 for Th1, 15 for Th17, 14 for Treg, 14 for lymphocyte exhaustion, and 7 for CTL) (Figure S4 and Table S2). Most of the genes with higher expression in NUCL have a central role in activating the pro-inflammatory response and inflammation (*TREM1*, *IL1RAP*, *NLRP3*, *CD14*, *NOD2*, *MEFV*, *LYN*). In contrast, genes with higher expression in VL are related to the complement system, inflammation and response to viruses (*C1QA*, *C1QB*, *HSP90AA1*, *TRIM10*).

### 3.6. In Vivo Cell-Mediated Immunity Confirms a Stronger Pro-Inflammatory Response in NUCL

**Figure 5:**
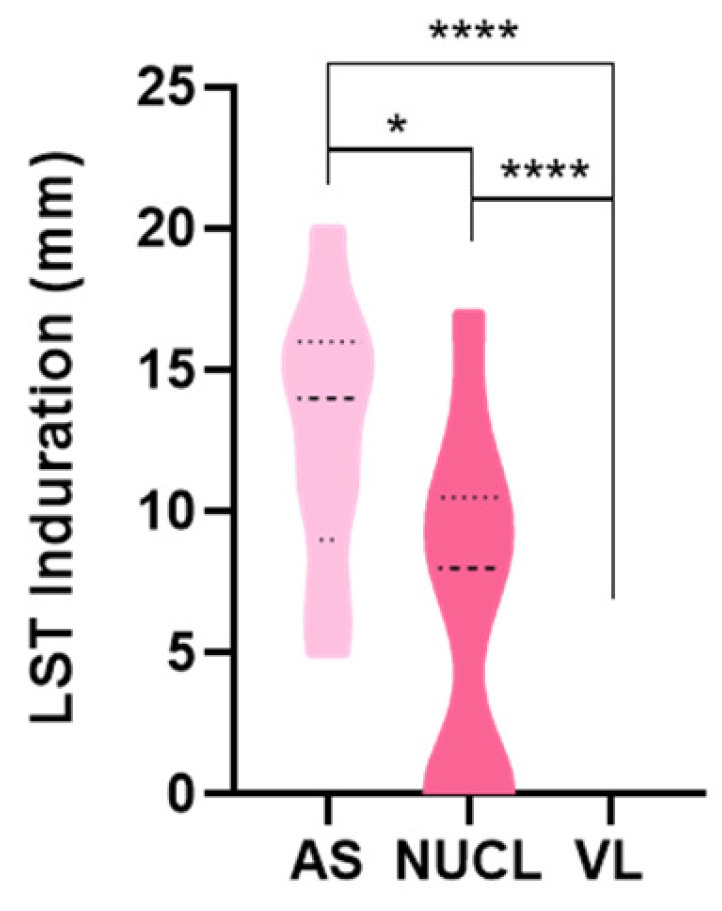
Comparison of induration of Leishmanin skin test (LST) lesion among asymptomatic (AS), non-ulcerated cutaneous leishmaniasis (NUCL) and visceral leishmaniasis (VL) individuals upon 72 h of intradermal injection of L. infantum chagasi antigen. Parametric test-*t* student and non-parametric Mann–Whitney. (*) *p*-value < 0.05, (****) *p*-value < 0.0001.

When the in vivo cell-mediated immunity (CMI) was assessed by Leishmanin Skin Test (LST) (Figure 5), we observed a significantly larger induration size in NUCL than in VL patients (*p* < 0.0001). The response was even higher in AS individuals. This finding reinforces the idea of proinflammatory immunity in NUCL as an intermediate immune state between AS infections and VL.

### 3.7. In Situ Cutaneous Evaluation Corroborates That NUCL Immune Response Is Predominantly Pro-Inflammatory

To validate the NUCL immunological profiles observed in the transcriptome, we compared counts of immunohistochemistry (IHC)-labeled cells between NUCL patients and negative controls (NEG). This analysis used individuals recruited and diagnosed as described in Sandoval Pacheco et al., 2023 [3]. We included the following cells/markers: macrophagic cells such as CD68, CD163, CD68/iNOS (M1), and CD163/IL-10 (M2); and dendritic cells CD1a (epidermal dendritic cell), CD1a/IL-12 (pro-inflammatory epidermal dendritic cell), CD1a/IL-10 (suppressor epidermal dendritic cell), CD11c (dermal dendritic cell), CD11c/IL-12 (pro-inflammatory dermal dendritic cell), CD11c/IL-10 (suppressor dermal dendritic cell), CD4^+^ T lymphocytes, CD4/IFN-γ (pro-inflammatory T helper lymphocyte), CD4/IL-10 (suppressor T lymphocyte), CD8^+^ T lymphocytes, CD8/IFN-γ (pro-inflammatory cytotoxic T lymphocyte), and CD8/IL-10 (suppressor cytotoxic T lymphocyte).

**Figure 6:**
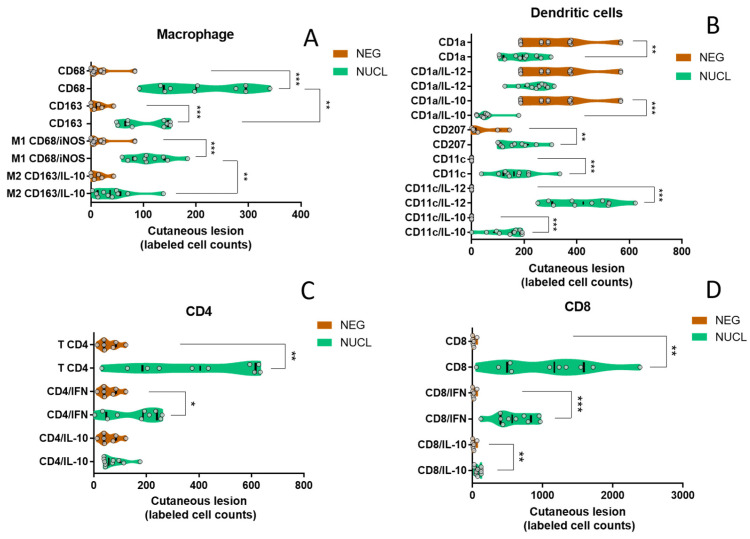
Comparison of the expression of antigen-presenting cells markers in cutaneous lesion between NUCL and NEG individuals. (**A**) The violin plots showing all points present comparison of in situ cutaneous counts of monocytes/macrophages (CD68 and CD163), macrophage cells M1 (CD68/iNOS) and M2 (CD163/IL-10). (**B**) Epidermal (CD1a), Langerhans (CD207), dermal (CD1c) dendritic cells, pro-inflammatory epidermal (CD1a/IL-12), suppressor epidermal (CD1a/IL-10), pro-inflammatory dermal (CD11c/IL-12), suppressor dermal (CD11c/IL-10). (**C**) pro-inflammatory CD4^+^ T lymphocytes (CD4/IFN-γ), suppressor T lymphocyte (CD4/IL-10). (**D**) pro-inflammatory CD8^+^ T helper lymphocytes (CD8/IFN-γ), suppressor T helper lymphocyte (CD8/IL-10) in in situ cutaneous lesion in NUCL (green) and healthy skin of NEG (brown) individuals by conventional and doble-staining immunohistochemistry (IHC). Parametric test-*t* student and non-parametric Mann–Whitney. (*) *p*-value < 0.05, (**) *p*-value < 0.01, (***) *p*-value < 0.001.

In the antigen-presenting cell subset, we observed an increase in macrophage CD68^+^ (*p* < 0.001), M1 (*p* < 0.001) in NUCL compared to NEG individuals. We also found an increase in CD68 in relation to CD163 (*p* < 0.01) and an increased M1 compared to M2 (*p* < 0.01) in the in situ cutaneous injury of NUCL individuals (Figure 6A). Likewise, we observed an increase in the cutaneous lesion of NUCL compared to NEG for the number of dermal dendritic cells CD11c (*p* < 0.001), pro-inflammatory dermal dendritic cells CD11c/IL-12 (*p* < 0.001) and suppressor dermal dendritic cells CD11c/IL-10 (*p* < 0.001). In contrast, for epidermal dendritic cells CD1a (*p* < 0.01) and suppressor epidermal dendritic cells CD1a/IL-10 (*p* < 0.001) there was a reduction in NUCL compared to the NEG skin (Figure 6B). A decrease in the count of epidermal dendritic cells expressing CD1a and the up-regulation of the *CD1A* gene observed in the bloodstream (Table S3) may be related to the migration of CD1a cells from cutaneous lesion to the bloodstream and/or a compensatory host response in overcome the CD1a inhibition caused by *L.* (*L.*) *infantum chagasi* in the early stages of infection and re-exposure. 

To the lymphocyte subset, a significant increase in the content of labeled cells in the cutaneous injury of the NUCL group compared to the skin of the NEG group was observed for CD4 (*p* < 0.01), CD4/IFN-γ (*p* < 0.05) (Figure 6C), CD8 (*p* < 0.01), CD8/IFN-γ (*p* < 0.001) and CD8/IL-10 (*p* < 0.01) (Figure 6D). The presented data point to the activation of a mixed immunity, with a predominant pro-inflammatory innate and adaptive responses concomitant with a mild regulatory response. That profile may be enough to lead to the control of the infection and prevent ulceration and the dissemination of the inflammatory response to other tissues and compartments of the host.

## 4. Discussion

We compared the transcriptomic profile in the bloodstream among patients with NUCL, VL, AS infections and negative control (NEG) individuals in order to identify cell-mediated immunity genes associated with different clinical outcomes. Also, we identified gene signatures of cell-mediated immunity in NUCL patients.

One of the initial challenges of our study was to find a type of tissue useful to compare the gene transcription of different forms of the disease, which, despite being caused by *L.* (*L.*) *infantum chagasi*, present an enormous topographic discrepancy of the lesions. While NUCL is restricted to the skin, VL spreads to tissues such as skin, the lymphatic system, including lymph nodes, bloodstream, liver, spleen, bone marrow, intestines, as well as lungs, and kidneys. We first assessed whether the blood gene expression of immune response profile was capable of reproducing the immune response previously described for NUCL skin lesions [19,20,21,52]. We demonstrated that the comparison of the blood gene expression of biomarkers of different immune response profiles (Th1, Th17, CTL, Treg and Th2) among NUCL, VL, and NEG partially reproduced the predominantly pro-inflammatory profiles of mixed Th1, Th17, but under the control of Treg and Th2 in NUCL lesions. These results confirmed that blood samples are useful for evaluating the immune response that occurs in NUCL lesions and allowed us to compare the immune response that occur in NUCL with the immune response in VL.

The functional aspects of the differentially expressed genes related to cell-mediated immunity in NUCL corroborate the idea that the four main mechanisms for controlling infection, maintaining a low parasite load, retention of the lesion localized in the skin site, the suppression of visceralization, and the absence of ulcers are as follows: (i) an efficient mechanism of innate immune response, (ii) migration and retention of leukocytes at the site of the skin lesion; (iii) the lymphocytes exhaustion as a mechanism for controlling the activation of adaptive immunity; and (iv) the control of oxidative stress in the host. On the other hand, the VL cases showed induction of proteases that play roles in the antigen cross-presentation pathway via MHC class I to CD8^+^ T lymphocytes and NK cells. That mechanism potentially activates both, cytotoxic and helper functions of CD8^+^ T lymphocytes leading to the T lymphocyte exhaustion. Furthermore, there was a suppression of the genes related to the mechanisms of innate immunity, migration and retention of leukocytes in peripheral tissues in VL cases.

### 4.1. T Lymphocyte Exhaustion

Both NUCL and VL are infections characterized by long term persistent infections. In chronic infections, depending on the infection load, the availability of pathogen antigens usually activates inhibitory receptors for the T lymphocyte response. It leads to a state of under activation known as exhaustion. The exhaustion state is characterized by reduced activation of phagocytes microbicidal mechanisms, which then favors the maintenance of a parasitic load widely described in cases of cutaneous and visceral leishmaniasis in human and canine hosts [18,34,53]. Interestingly, here, we presented significantly increased expression of exhaustion biomarkers (*PDCD1*, *PDCD1LG2*, *LAG3*) in both NUCL and VL when compared to the NEG group (Figure S3). However, many biomarkers of lymphocyte exhaustion were DEG’s exclusively in NUCL (*PDCD1[PD1]*, *LAG3*) or with higher expression in NUCL than in VL (*CD274*, *KLRC1*, *KLRD1*). The Log2FC value of the *PDCD1*, *PDCD1LG2*, *LAG3* genes was not significant for VL due to the high data variability in this group (Figure S3).

The immune response at the NUCL in situ skin lesion remains predominantly pro-inflammatory, characterized by increased counts of leukocytes expressing pro-inflammatory mediators (Figure 6) and positive LST (Figure 5). On the other hand, the lymphocyte exhaustion may be involved in inhibition of several mediators of Th1, Th17, CTL, Treg and Th2 responses and their receptors in the bloodstream (Figure S2). Comparatively, the suppressive effect of lymphocyte exhaustion on mediators of immune response seems to be even more intense in the bloodstream of VL patients. The immune response profiles were significantly suppressed in the VL vs. NEG comparison (Figure S2, Table S2). Compared to the NEG group, the expression of the immune response biomarkers was significantly suppressed in VL in 16/25 (64%) of the Th1, 7/9 (77%) of CTL, 13/18 (72%) of Th17, 15/17 (88%) of the Treg, 20/24 (83%) of Th2 biomarkers. This is potentially a causal basis for the greater severity of the clinical condition in VL. In contrast, the IL-10 levels remained high in the VL, even with lower expression of *FOXP3* (Figure S2D) and *IL2RA* (CD25) (Figure 4). The high level of IL-10 associated with lower expression of *FOXP3* and *IL2RA* is consistent with a type 1 peripheral regulatory (Tr1) response as a source of IL-10 in VL, rather than the thymic Treg. The role of the Tr1 response in maintaining IL-10 levels has been described in human VL, chronic CL and canine VL [18,54,55].

### 4.2. Innate IMMUNE Response

The effective action of innate immunity in NUCL lesion appears to be affected by several factors: the up-regulation of innate immune response genes (Figure S4); the production of proinflammatory cytokines (IFN-γ, IL-12, IL-17); the cell migration mediators (*VCAM1*, *ICAM5*, *CXADR*); and a greater preservation of factors related to leukocyte infiltration in peripheral tissues (*SERPINA10*, *TREM1*). The gene for the protease inhibitor SERPINB10 was identified as a candidate gene in a GWAS for typical CL caused by *L. braziliensis* [56]. Despite this, this action of innate immunity in the NUCL in situ lesions is not capable of promoting the clearance of the infection. Consistent with this picture, we detected the down-regulation of DEGs involved in the acidification of intracellular compartments and microbicidal mechanisms of phagocytes (*LPO*, *MPO*, *ATP6V0E1*, *ATP6V1E1*, *LTF*). The down-regulation of those genes would lead to the prevention of oxidative stress, of tissue damage such as ulceration of the infected skin, as well as keeping a permissive environment for the long-term parasite survival. Indeed, NUCL is characterized by a low parasite load in the papular skin lesion. Conversely, *MPO* has been reported to be highly up-regulated in clinic canine VL [18]. Apparently VL cases present greater difficulty in the infiltration of leukocytes in the peripheral lesions. For example, the negative LST test (Figure 5) associated with the strong down-regulation of *SERPINA10* and *TREM1* in VL individuals.

### 4.3. CD8^+^ T Lymphocytes

Cytokine production and the cytotoxicity of CD8^+^ T lymphocytes have been described as protective in VL [57] and in typical CL [58]. Here, we observed an increase in IFN-γ producing T CD8^+^ in the NUCL skin lesion. Likewise, there was an increased expression of *GZMA*, *GNLY* and TNF in the bloodstream, and up-regulation of a network of proteases of the cross-presentation pathway in VL. This set of data corroborates the idea of a protective role of CD8^+^ T lymphocytes in NUCL [21] and reinforces the cross-presentation of antigens via MHC class I as the main mechanism of CD8^+^ T activation in VL due to *L.* (*L.*) *infantum chagasi*.

Genes grouped in the same co-expression module can act together or be regulated at similar levels. The co-expression modules somehow confirmed the discrepancies in the bloodstream gene expression between NUCL and VL already shown in unsupervised analysis (Figure 2). While the modules enriched in VL have a higher NES value, the module enriched in NUCL had a lower NES value (Figure S6). Another noteworthy finding is that the genes from the modules enriched in both NUCL and VL also enriched pathways related to the regulatory interaction between lymphoid and non-lymphoid cells. This scenario is compatible with both the action of a Tr1-type peripheral regulatory response, lymphocyte exhaustion and its suppressive effects on immune response in both NUCL and VL.

Another pathway enriched in the module related to NUCL was neutrophil granulation. The highlight of this pathway may be related to a down-regulation of oxidative stress DEGs such as *MPO* and *LPO*. These genes are potentially involved in tissue damage by necrosis and ulceration (absent in NUCL). The hub genes of the co-expression modules protein interaction networks are also directly or indirectly related to the regulations of innate and adaptive immunity by promoting inflammation in NUCL and VL, and chromatin regulation.

The three methods employed here—the gene signatures of CMI in NUCL; the LST; and the immunohistochemical profile of NUCL skin lesions—corroborated and pointed out that the CMI in NUCL is associated with a benign proinflammatory condition, which is intermediate between the asymptomatic infection (AS) and the susceptibility pole (VL). A broader exploration of such molecular targets will open the way for new therapeutic and immunomodulatory strategies for severe and anergic forms of leishmaniasis, such as VL and diffuse cutaneous leishmaniasis. Concomitantly, the transcriptomic differences reported here suggest a genomic study of the parasite, of the host, of the vector, and the skin microbiota of hosts residing in areas where NUCL is endemic. Further studies may expand understanding of the balance of genetic variability that leads to greater adaptation between the parasite and the host in NUCL.

## Figures and Tables

**Table 1:** LogFC value of the expression in NUCL cases related to the expression in AS, VL, NEG groups, and function for each of the 8 DEGs identified as gene signature of cell-mediated immunity in NUCL.

CMI DEG	LogFC_AS_	LogFC_VL_	LogFC_NEG_	Function
*BATF2*	1.25	NS	NS	Enhances CD8^+^ T-cell infiltration and activation.
*SH3PXD2B*	1.15	−1.77	4.49	Allows NOX1- or NOX3-dependent reactive oxygen species (ROS) generation.
*CD274*	1.10	NS	0.99	Ligand for the inhibitory receptor PDCD1/PD-1.
*GBP1*	0.84	−1.41	NS	Inflammasome assembly in response to infection.
*CCR3*	0.83	5.92	1.89	Receptor for C-C type chemokine.
*CR1*	0.8	1.32	NS	Receptor that plays a critical role in the capture and clearance of complement-opsonized pathogens.
*MARCO*	−1.13	−1.46	6.62	Pattern recognition receptor (PRR) which binds bacteria.
*COLEC12*	−1.61	NS	3.37	Scavenger receptor correlated to infiltration of suppressor immune cells.

NUCL—non-ulcerated cutaneous leishmaniasis, VL—visceral leishmaniasis, AS—asymptomatic, NEG—non-infected. LogFC_AS_—Log fold-chand value of NUCL vs. AS, LogFC_VL_—Log fold-chand value of NUCL vs. VL, NS—non-significant LogFC (*p* > 0.05).

## Data Availability

The raw data supporting the conclusions of this article will be made available by the authors on request. The original contributions presented in this study are included in the article/Supplementary Materials. Further inquiries can be directed to the corresponding author.

## Supplementary Materials

The following supporting information can be downloaded at: https://www.mdpi.com/article/10.3390/microorganisms13020413/s1, Figure S1. Functional enrichment of gene expression for the datasets NUCL vs. NEG (A and B) and VL vs. NEG (C and D). Figure S2. Comparison of the expression of the adaptative immune responses Th1 (A), CTL (B), Th17 (C), Treg (D), Th2 (E) biomarkers in blood samples among NUCL, VL and NEG individuals. Figure S3. Comparison of Exhaustion of T-lymphocyte biomarkers among groups NUCL (blue), VL (red), NEG (gray) individuals. Figure S4. Comparison of innate immune response biomarkers among groups NUCL (blue), VL (red), NEG (gray) individuals. Genes grouped in the same co-expression module can act together or be regulated at similar levels. File S1. CO-EXPRESSION_MODULES_ANALYSIS: we performed co-expression analysis to identify co-expression module more enriched for each clinical group [59,60,61,62,63,64,65,66,67,68,69,70]. Figure S5. Correlation among co-expression modules and count of cell phenotype markers in cutaneous injury in NUCL patients. Figure S6. Intensity and sense of expression for co-expression modules in each group. Figure S7. Functional enrichment analysis for co-expression modules M1 (A), M2 (B), M4 (C), M5 (D). Figure S8. Interaction network between the genes comprising the co-expression modules M1, M2, M3, M4 and M5. Table S1. Clinical outcome, origin—location of residence, number of lesions, term since symptoms manifestation, result in serological diagnoses (ELISA) for Immunoglobulin G (IgG) and IgM and size of lesion in Delayed-type hypersensitivity (DTH) of participants. Table S2. Statistically different differential expression between genes coding for biomarkers of Th1, Th2, Th17, CTL, Treg immune responses profiles, T lymphocyte exhaustion and innate immune response. Table S3. Up-regulated differently expressed genes (DEGs) related to cell-mediated immunity (CMI) exclusive in NUCL or in VL and DEGs commonly expressed in NUCL and VL were selected from enriched pathways functionally related to CMI for the datasets NUCL vs. NEG and VL vs. NEG. Table S4. Down-regulated differently expressed genes (DEGs) related to cell-mediated immunity (CMI) exclusive in NUCL or in VL and DEGs commonly expressed in NUCL and VL were selected from enriched pathways functionally related to CMI for the datasets NUCL vs. NEG and VL vs. NEG.
