# Supplementary material for: Molecular Insights into Cell-Mediated Immunity in Atypical Non-Ulcerated Cutaneous Leishmaniasis
Source: Microorganisms. 2025 Feb 13;13(2):413. doi: 10.3390/microorganisms13020413 (PMC11858551; doi:10.3390/microorganisms13020413)
Supplement: Supplementary file 1 [file microorganisms-13-00413-s001.zip › File S1. CO-EXPRESSION_MODULES_ANALYSIS.pdf]

S1.1 - Correlation between immune cell phenotypes in NUCL cutaneous lesion and co-expression modules in the blood

Genes grouped in the same co-expression module can act together or be regulated at similar levels. The genes expressed in the different groups of this study were grouped into 5 modules based on their expression levels (M1, M2, M3, M4, M5 co-expression modules). To identify the co-expression modules more enriched in the NUCL immune response, data from the count of cell phenotypes present *in situ* cutaneous (NUCL and NEG, Fig 6), were used to test the correlation with the average fold change count of each 5 co-expression modules. The modules M1 (inversely correlated), M2, M4 and M5 (directly correlated) were the modules more significantly correlated with the count of cell phenotype subsets (Fig. S5).

S1.2 - Co-expression modules pathways enrichment analysis

The co-expression module more enriched in the NUCL group (Fig. S6) was the M2 (NES = 2.24, $P_{adjusted} < 0.001$). The pathways more enriched by the co-expression module M2 were “Immunoregulatory Interactions between a Lymphoid and a non-Lymphoid cell” and “Neutrophil degranulation” (Fig. S7), indicating activation of the innate immunity and the regulation of the triggering adaptative immune response in NUCL. Otherwise, the Modules M3 (NES = 4.45, $P_{adjusted} = 0.00$) and M1 (NES = -3.54, $P_{adjusted} = 0.00$) were more enriched in VL cases (Fig. S6). Pathway enrichment for the Module M1 includes the pathways “Interferon Signaling”, “Signaling by Interleukins”, “Fcgamma receptor (FCGR) dependent phagocytosis”, “Fc epsilon receptor (FCERI) signaling” and “Immunoregulatory Interactions between a Lymphoid and a non-Lymphoid cell” (Fig. S7). No significant pathway ($P_{adjusted} < 0.05$) was enriched for the Module 3.

CEMiTool also integrates coexpression analysis with protein–protein interaction data. The protein-protein interaction networks hub genes were taken as a reference to infer

the functional context of each co-expression module, although no co-expression hub gene stood out as being central in terms of interaction intensity in each module (Fig. S8). The gene *MEFV* innate immunity regulator was down-regulated in NUCL individuals and stands out in module 1 (Fig. S8A) as it encodes a pyrin that acts as an autophagy receptor for the degradation of several inflammasome components, including CASP1, NLRP1 and NLRP3, hence preventing excessive IL-1β and IL-18-mediated inflammation [1,2]. In module 2, the hub genes *MATK, PADI4* were highlighted (Fig. S8B). The *MATK* encodes the Megakaryocyte-Associated Tyrosine Kinase is a nonreceptor tyrosine kinase that negatively regulates the activity of Src family protein tyrosine kinases and may play an inhibitory role in the control of T-cell proliferation [3]. Peptidyl Arginine Deiminase 4 (*PADI4*) promotes profound chromatin decondensation during the innate immune response to infection in neutrophils by mediating formation of H1R54ci, required for the formation of neutrophil extracellular traps (NETs) [4,5]. In Module 4 (M4) (Fig. S8D) the gene hubs *GIMAP1* - GTPase, IMAP Family Member 1 wich is involved in the differentiation of T helper (Th) cells of the Th1 lineage, and the related mouse gene has been shown to be critical for the development of mature B and T lymphocytes [6,7]. Module 5 (M5) (Fig. S8E) includes *SMARCE1, MALT1, NKTR, MICA, HLA-DOB.* SWI/SNF Related, Matrix Associated, Actin Dependent Regulator of Chromatin, Subfamily E, Member 1 (*SMARCE1)* is involved in transcriptional activation and repression of select genes by chromatin remodeling and intratumoral infiltration of $CD8^+$ T cell in ovarian tumors [8]. MALT1 Paracaspase is a caspase-like protease that plays a role in BCL10-induced activation of NF-kappaB. NF-κB signaling and concomitant activation, proliferation and cytokine response in T and B cells relies on MALT1 [9]. *NKTR* encodes a membrane-anchored protein component of a putative tumor-recognition complex involved in the function of NK cells [10]. *MICA* encodes the highly polymorphic major histocompatibility complex class I chain-related protein A that acts as a stress-induced self-antigen that is recognized by gamma delta T-cells [11]. HLA-DOB is an

important modulator in the HLA class II restricted antigen presentation pathway by interaction with the HLA-DM molecule in B-cells [12].

References

1. Papin S, Cuenin S, Agostini L, Martinon F, Werner S, Beer HD, Grütter C, Grütter M, Tschopp J. The SPRY domain of Pyrin, mutated in familial Mediterranean fever patients, interacts with inflammasome components and inhibits proIL-1beta processing. Cell Death Differ. 2007 Aug;14(8):1457-66. doi: 10.1038/sj.cdd.4402142.
2. Kimura T, Jain A, Choi SW, Mandell MA, Schroder K, Johansen T, Deretic V. TRIM-mediated precision autophagy targets cytoplasmic regulators of innate immunity. J Cell Biol. 2015 Sep 14;210(6):973-89. doi: 10.1083/jcb.201503023.
3. Lee BC, Avraham S, Imamoto A, Avraham HK. Identification of the nonreceptor tyrosine kinase MATK/CHK as an essential regulator of immune cells using Matk/CHK-deficient mice. Blood. 2006 Aug 1;108(3):904-7. doi: 10.1182/blood-2005-12-4885.
4. Wang Y, Wysocka J, Sayegh J, Lee YH, Perlin JR, Leonelli L, Sonbuchner LS, McDonald CH, Cook RG, Dou Y, Roeder RG, Clarke S, Stallcup MR, Allis CD, Coonrod SA. Human PAD4 regulates histone arginine methylation levels via demethylimination. Science. 2004 Oct 8;306(5694):279-83. doi: 10.1126/science.1101400.
5. Neeli I, Khan SN, Radic M. Histone deimination as a response to inflammatory stimuli in neutrophils. J Immunol. 2008 Feb 1;180(3):1895-902. doi: 10.4049/jimmunol.180.3.1895.
6. Webb LM, Datta P, Bell SE, Kitamura D, Turner M, Butcher GW. GIMAP1 Is Essential for the Survival of Naive and Activated B Cells In Vivo. Immunol. 2016 Jan 1;196(1):207-16. doi: 10.4049/jimmunol.1501582.

7. Datta P, Webb LM, Avdo I, Pascall J, Butcher GW. Survival of mature T cells in the periphery is intrinsically dependent on GIMAP1 in mice. Eur J Immunol. 2017 Jan;47(1):84-93. doi: 10.1002/eji.201646599.
8. Giannakakis A, Karapetsas A, Dangaj D, Lanitis E, Tanyi J, Coukos G, Sandaltzopoulos R. Overexpression of SMARCE1 is associated with CD8+ T-cell infiltration in early stage ovarian cancer. Int J Biochem Cell Biol. 2014 Aug;53:389-98. doi: 10.1016/j.biocel.2014.05.031.
9. Meininger I, Krappmann D. Lymphocyte signaling and activation by the CARMA1-BCL10-MALT1 signalosome. Biol Chem. 2016 Dec 1;397(12):1315-1333. doi: 10.1515/hsz-2016-0216.
10. Anderson SK, Gallinger S, Roder J, Frey J, Young HA, Ortaldo JR. A cyclophilin-related protein involved in the function of natural killer cells. Proc Natl Acad Sci U S A. 1993 Jan 15;90(2):542-6. doi: 10.1073/pnas.90.2.542.
11. Wu J, Groh V, Spies T. T cell antigen receptor engagement and specificity in the recognition of stress-inducible MHC class I-related chains by human epithelial gamma delta T cells. J Immunol. 2002 Aug 1;169(3):1236-40. doi: 10.4049/jimmunol.169.3.1236.
12. Fallas JL, Tobin HM, Lou O, Guo D, Sant'Angelo DB, Denzin LK. Ectopic expression of HLA-DO in mouse dendritic cells diminishes MHC class II antigen presentation. J Immunol. 2004 Aug 1;173(3):1549-60. doi: 10.4049/jimmunol.173.3.1549.
