# Supplementary material for: Molecular Insights into Cell-Mediated Immunity in Atypical Non-Ulcerated Cutaneous Leishmaniasis
Source: Microorganisms. 2025 Feb 13;13(2):413. doi: 10.3390/microorganisms13020413 (PMC11858551; doi:10.3390/microorganisms13020413)
Supplement: Supplementary file 1 [file microorganisms-13-00413-s001.zip › SUPPLEMENTARY_FIGURES_MANUSCRIPT_RNA-seq_NUCL_FINAL.pdf]

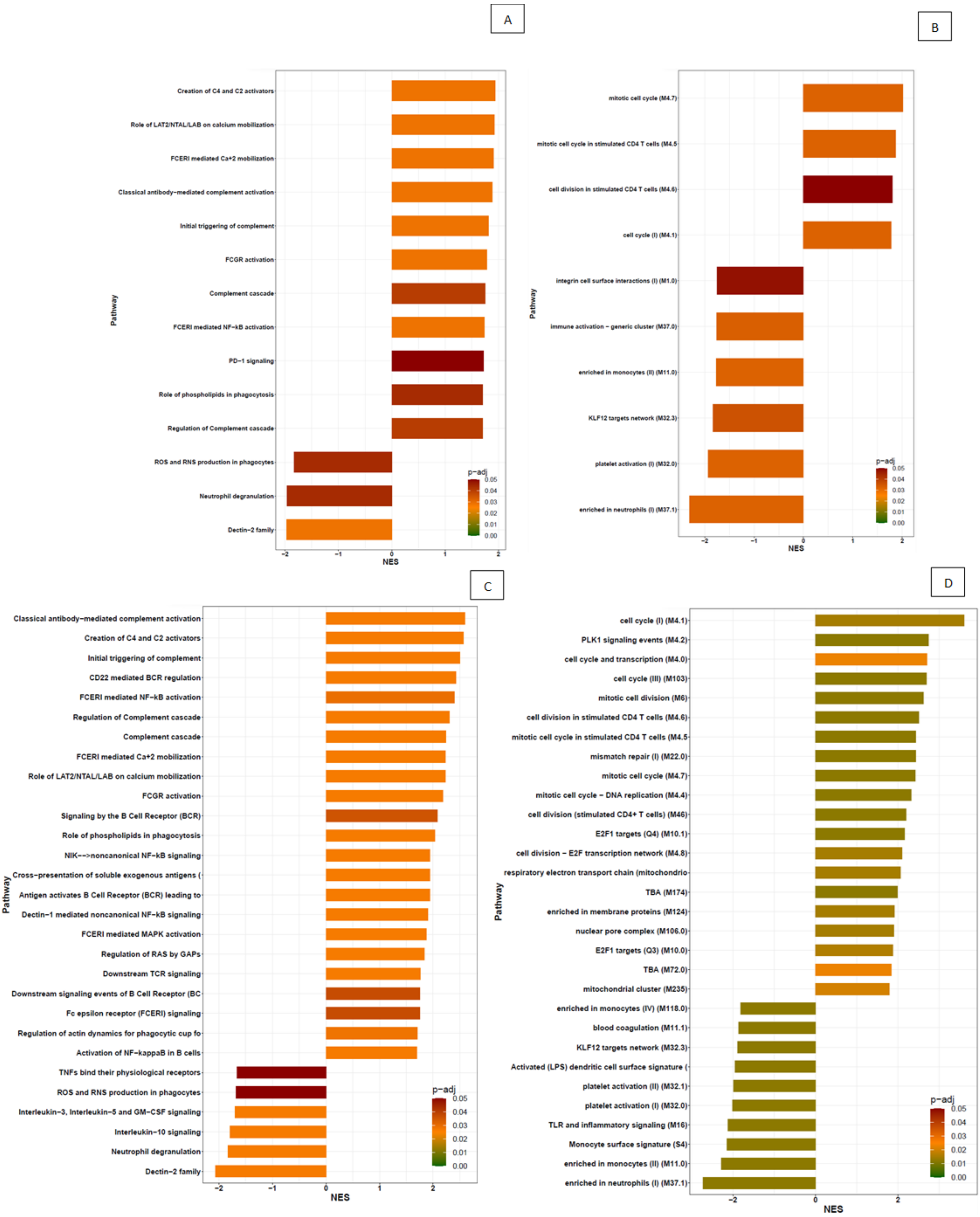

Figure S1. Functional enrichment of gene expression for the contrasts NUCL X NEG (A and B) and VL X NEG (C and D). Differently expressed genes DEGs were grouped in the functional enrichment analysis using the Reactome Immune System (RIS) - A and C, and Blood Transcription Modules (BTM) - B and D databases. Pathways related to cell-mediated immune (CMI) response with statistical significance ($P_{adjusted}$ < 0.05) were priorized for search of CMI DEGs in NUCL and VL.

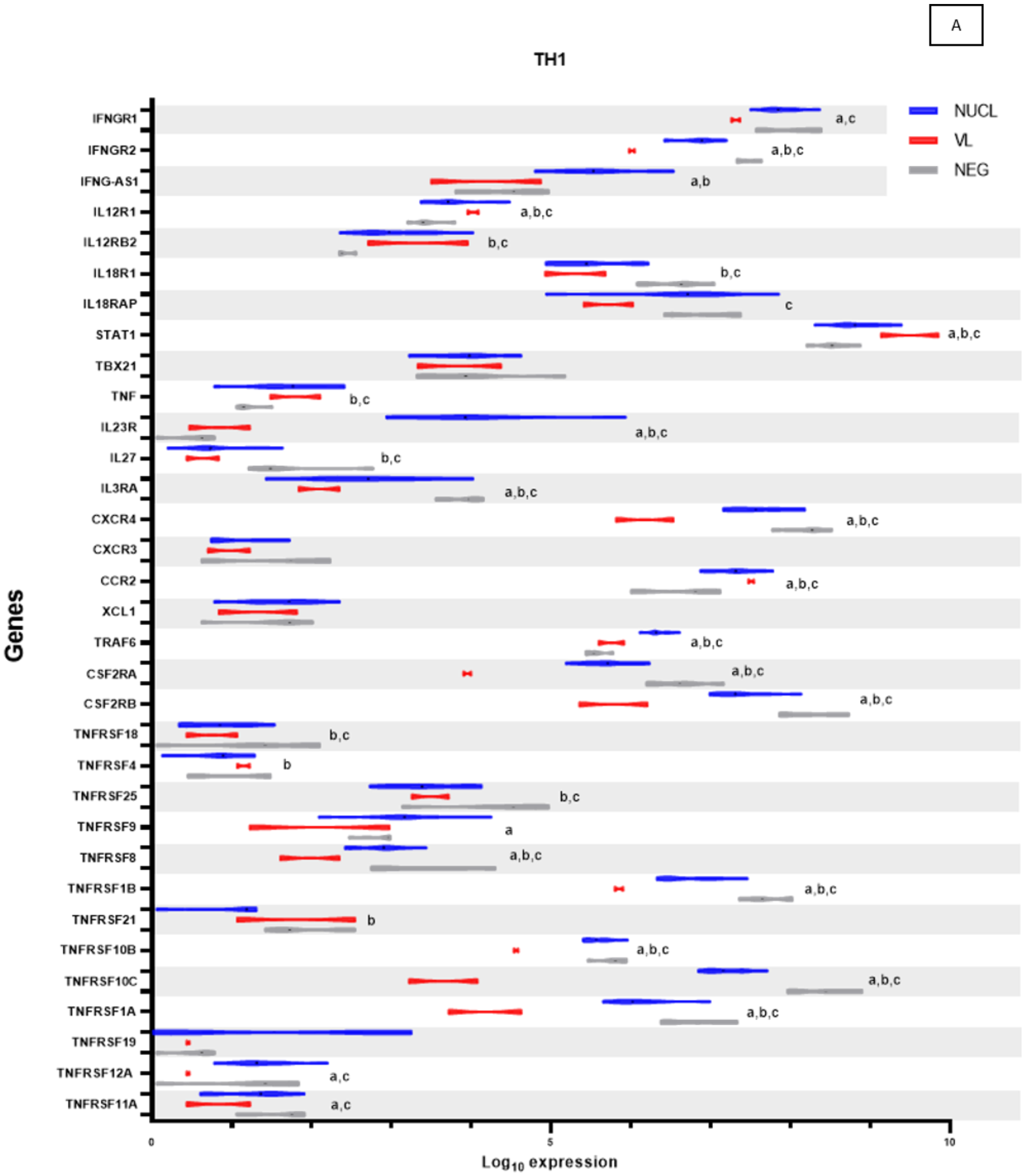
A
TH1
NUCL
VL
NEG
Genes
IFNGR1
IFNGR2
IFNG-AS1
IL12R1
IL12RB2
IL18R1
IL18RAP
STAT1
TBX21
TNF
IL23R
IL27
IL3RA
CXCR4
CXCR3
CCR2
XCL1
TRAF6
CSF2RA
CSF2RB
TNFRSF18
TNFRSF4
TNFRSF25
TNFRSF9
TNFRSF8
TNFRSF1B
TNFRSF21
TNFRSF10B
TNFRSF10C
TNFRSF1A
TNFRSF19
TNFRSF12A
TNFRSF11A
a,c
a,b,c
a,b
a,b,c
b,c
b,c
c
a,b,c
b,c
a,b,c
b,c
a,b,c
a,b,c
a,b,c
a,b,c
a,b,c
a,b,c
b,c
b
b,c
a
a,b,c
a,b,c
b
a,b,c
a,b,c
a,b,c
a,c
a,c
0
5
10
Log10 expression

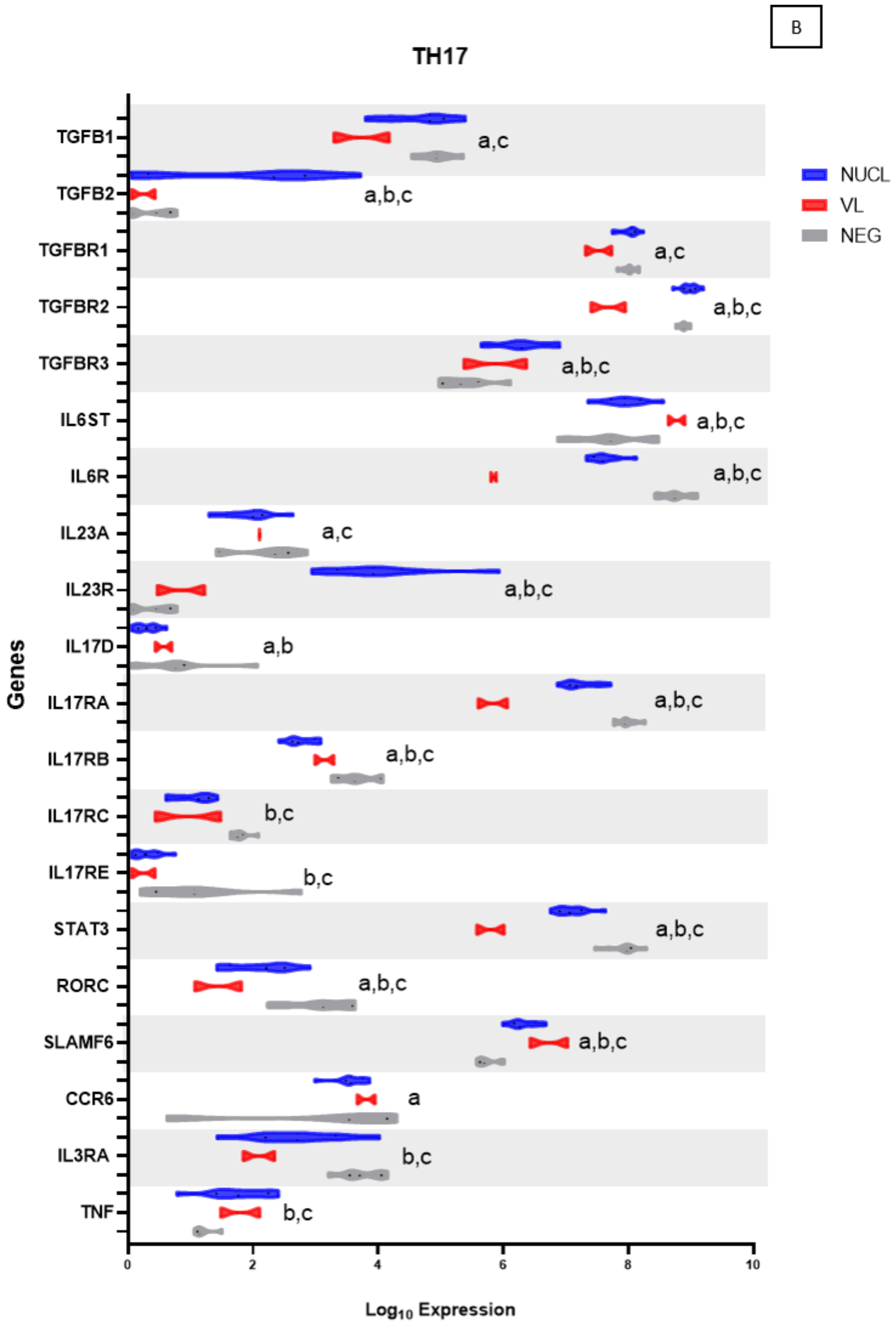

B
TH17
NUCL
VL
NEG
Genes
TGFB1
TGFB2
TGFBR1
TGFBR2
TGFBR3
IL6ST
IL6R
IL23A
IL23R
IL17D
IL17RA
IL17RB
IL17RC
IL17RE
STAT3
RORC
SLAMF6
CCR6
IL3RA
TNF
a,c
a,b,c
a,c
a,b,c
a,b,c
a,b,c
a,b,c
a,c
a,b,c
a,b
a,b,c
a,b,c
b,c
b,c
a,b,c
a,b,c
a,b,c
a
b,c
b,c
0
2
4
6
8
10
Log10 Expression

C

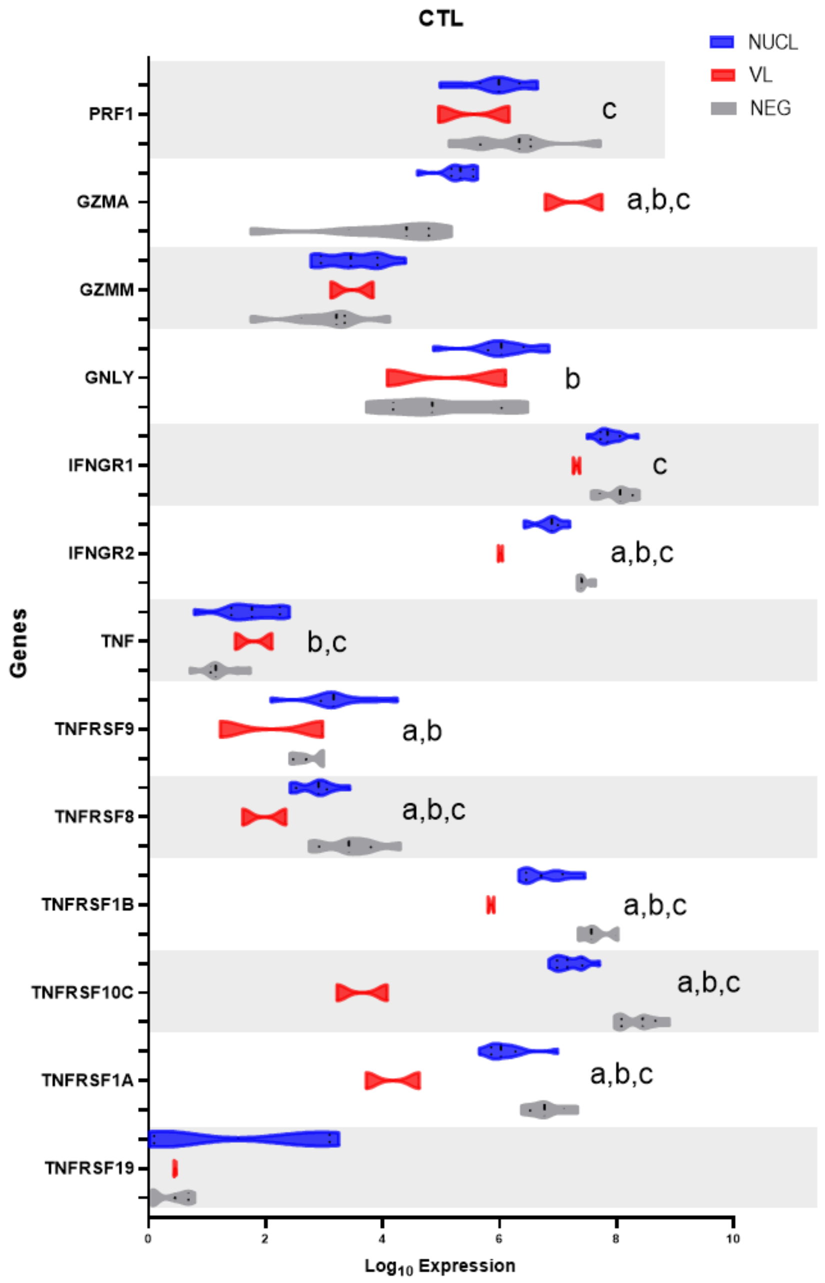
CTL
NUCL
VL
NEG
PRF1
c
GZMA
a,b,c
GZMM
GNLY
b
IFNGR1
c
IFNGR2
a,b,c
TNF
b,c
TNFRSF9
a,b
TNFRSF8
a,b,c
TNFRSF1B
a,b,c
TNFRSF10C
a,b,c
TNFRSF1A
a,b,c
TNFRSF19
Genes
0
2
4
6
8
10
Log10 Expression

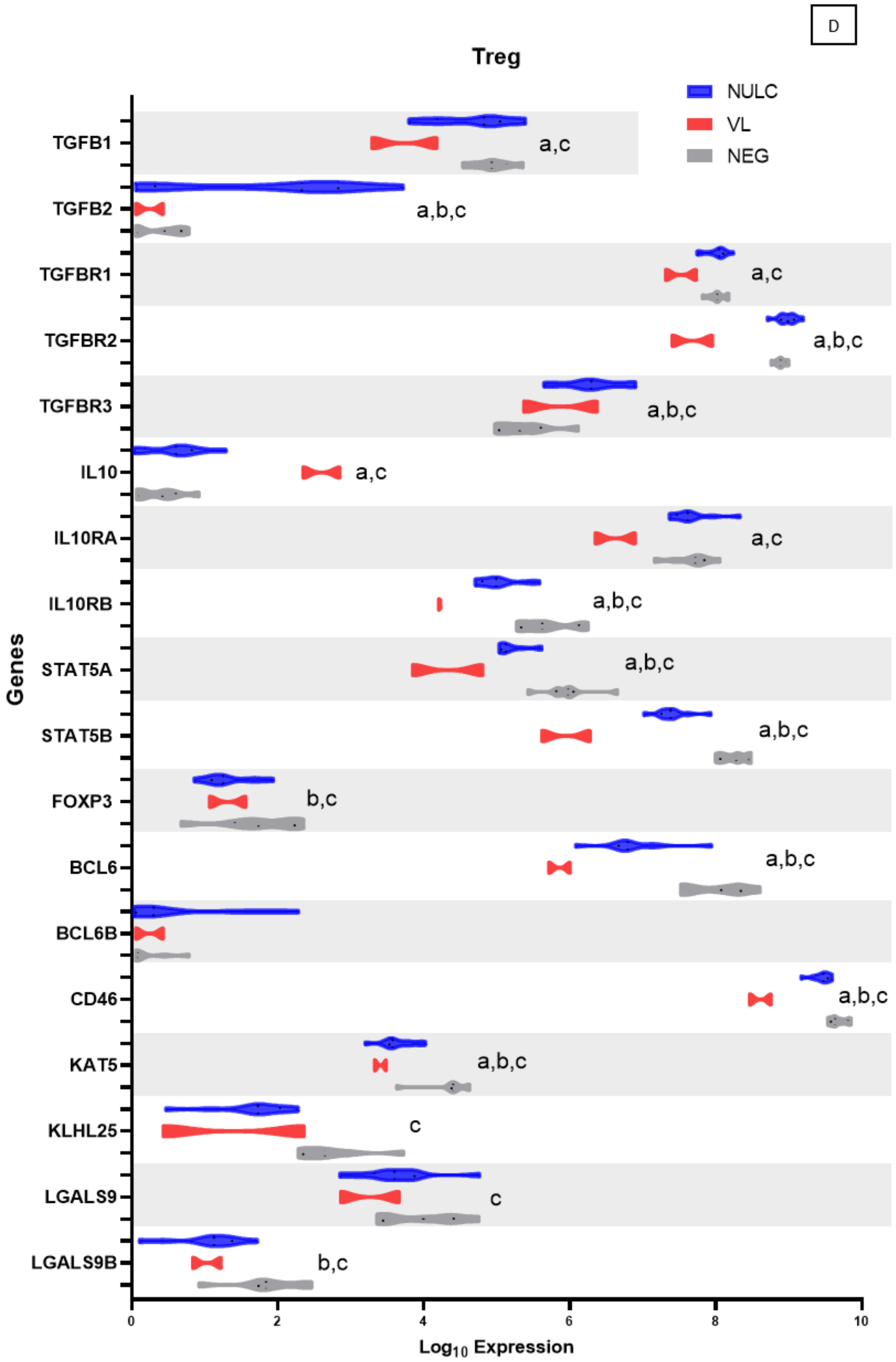
D
Treg
NULC
VL
NEG
TGFB1
a,c
TGFB2
a,b,c
TGFBR1
a,c
TGFBR2
a,b,c
TGFBR3
a,b,c
IL10
a,c
IL10RA
a,c
IL10RB
a,b,c
STAT5A
a,b,c
STAT5B
a,b,c
FOXP3
b,c
BCL6
a,b,c
BCL6B
CD46
a,b,c
KAT5
a,b,c
KLHL25
c
LGALS9
c
LGALS9B
b,c
Genes
0
2
4
6
8
10
Log10 Expression

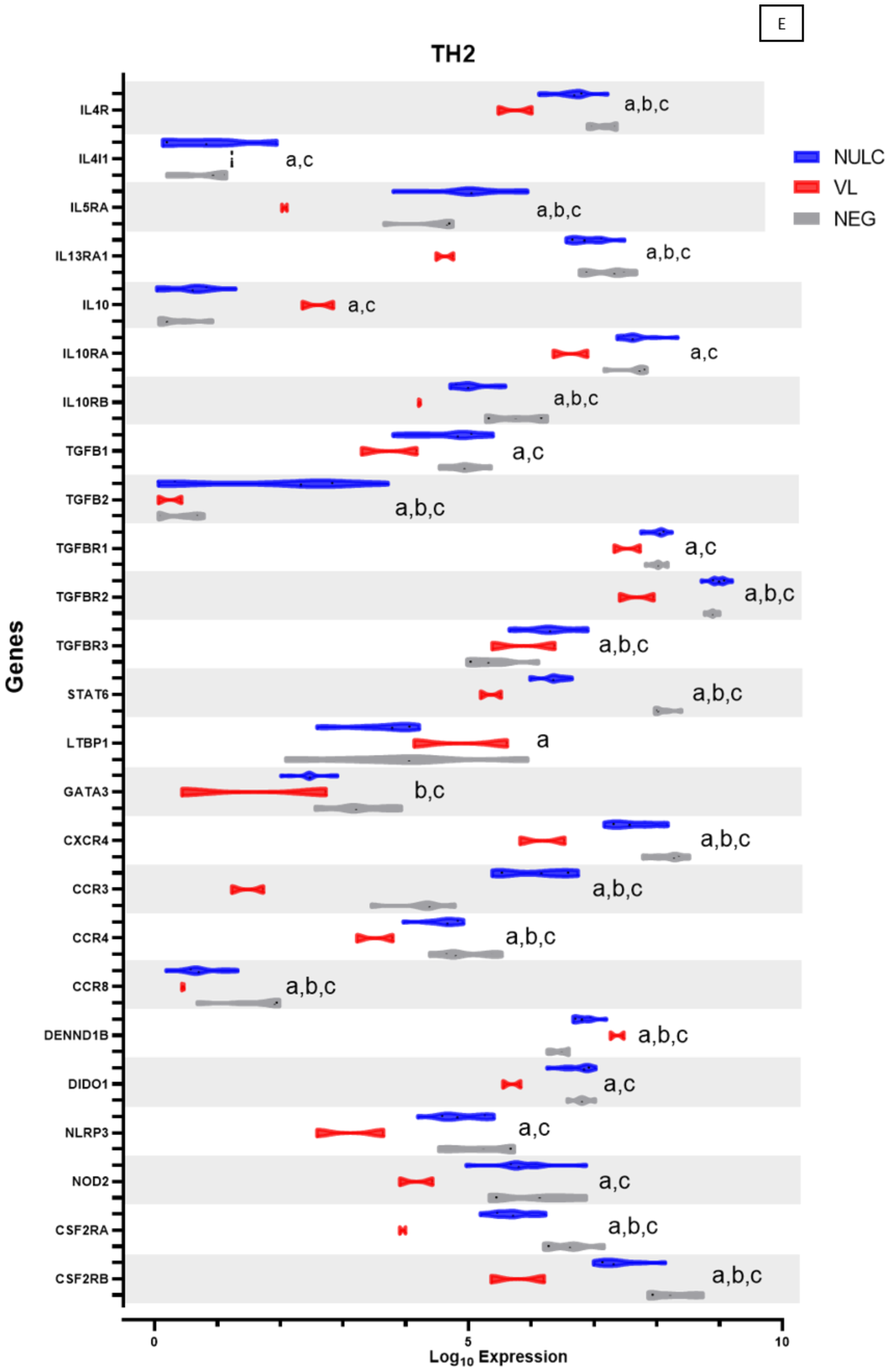

Figure S2. Comparison of the expression of the adaptative immune responses Th1 (A), CTL (B), Th17 (C), Treg (D), Th2 (E) biomarkers in blood samples among NUCL, VL and NEG individuas. The Log expression values of the imune response biomarkers were compared among NUCL (blue), VL (red) and NEG (gray) individuas. Lists of biomarkers were searched in databases such as InnateDB, MGI and in literature related to immune response profiles. Biomarkers with apparent differences in expression between groups were selected for comparison between groups. Parametric test-T student and non-parametric Mann-Whitney, (a) significant statistical difference between NUCL and VL, (b) significant statistical difference between NUCL and NEG, (c) significant statistical difference between VL and NEG.

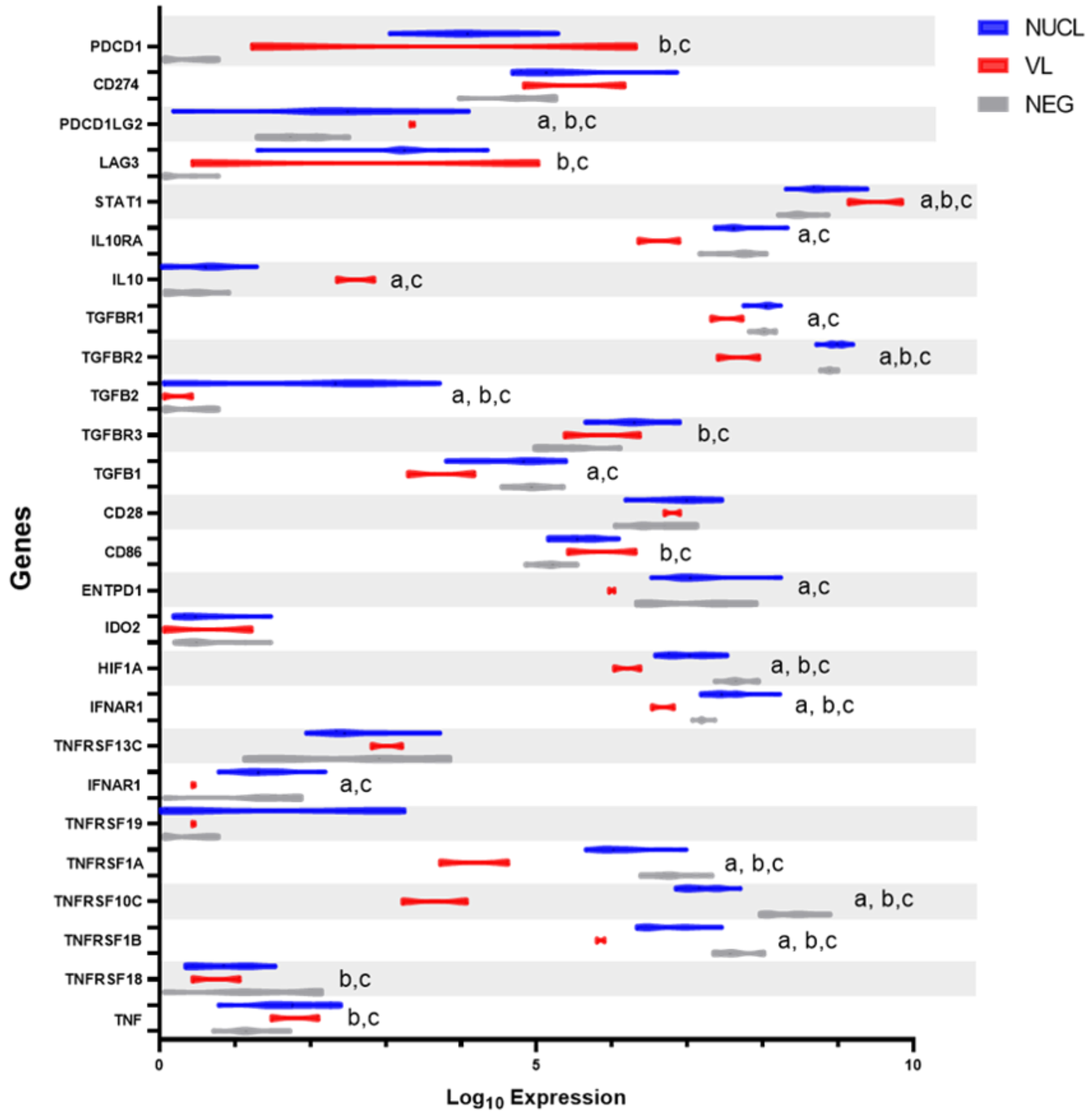

Figure S3. Comparison of Exhaustion of T-lymphocyte biomarkers among groups NUCL (blue), VL (red), NEG (gray) individuals. List of Exhaustion of T-lymphocyte biomarkers were searched in literature related to T-cell exhaustion. Biomarkers with apparent differences in expression between groups were selected for comparison between groups. Parametric test-T student and non-parametric Mann-Whitney, (a) statistical difference between NUCL and VL, (B) statistical difference between NUCL and NEG, (C) statistical difference between VL and NEG.

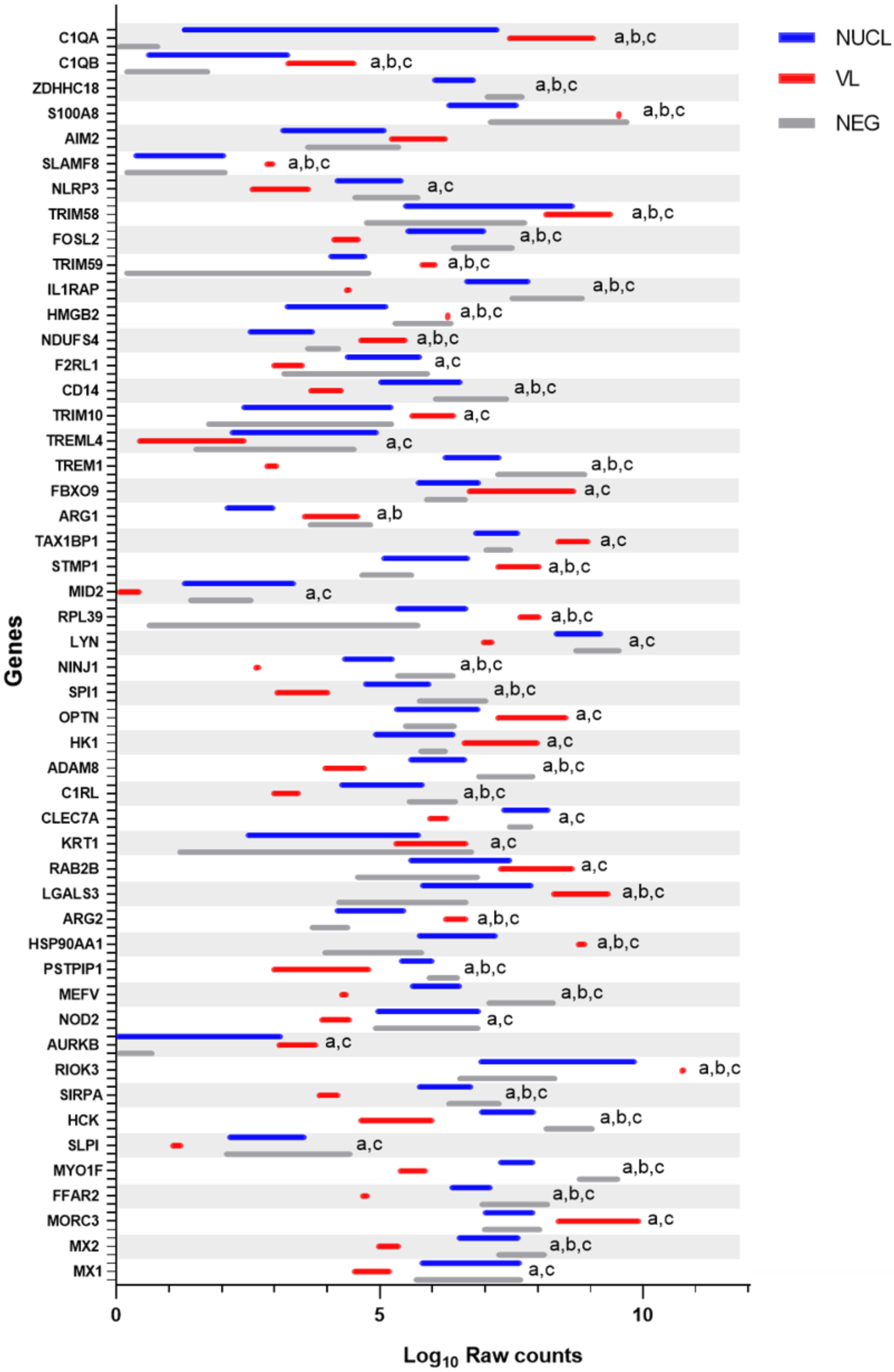

Figure S4. Comparison of innate imune response biomarkers among groups NUCL (blue), VL (red), NEG (gray) individuals. List of innate imune biomarkers were searched in literature related to innate imune response. Biomarkers with apparent differences in expression between groups were selected for comparison between groups. Parametric test-T student and non-parametric Mann-Whitney, (a) statistical difference between NUCL and VL, (B) statistical difference between NUCL and NEG, (C) statistical difference between VL and NEG.

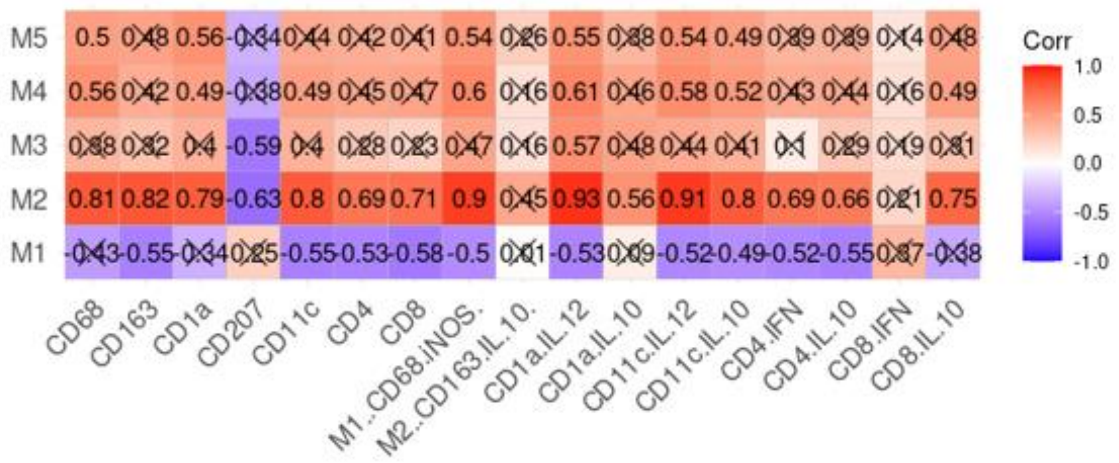

Figure S5. Correlation among co-expression modules and count of cell phenotypes martkers in cutaneous infury in NUCL patients. The convencional and doble-marked IHQ absolut counts were emploied in order to identified blood transcriptomic co-expression module correlated with the pattern of inflammatory infiltrate in NUCL injury. Positive correlation is represented in red. Negative correlation is represented in blue. Non-signinificant correlation p > 0.05. Pearson correlogram were plotted in R.

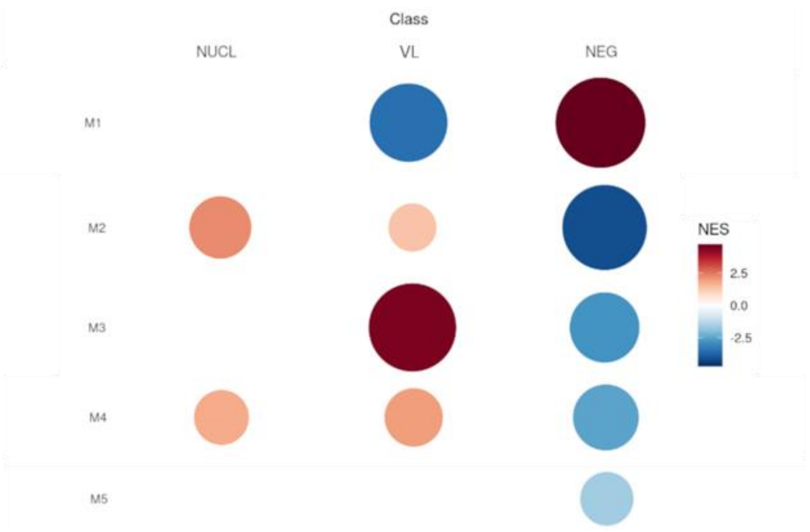

Figure S6. Intensity and sense of expression for co-expression modules in each group. The expression of each co-expression module is represented for dots in which the size of the dot varies according to the intensity of the expression. Red dots represents positive Normalized Enrichment Score (NES). Blue dots representes negative NES. Empty spots represent non-significant correlation.

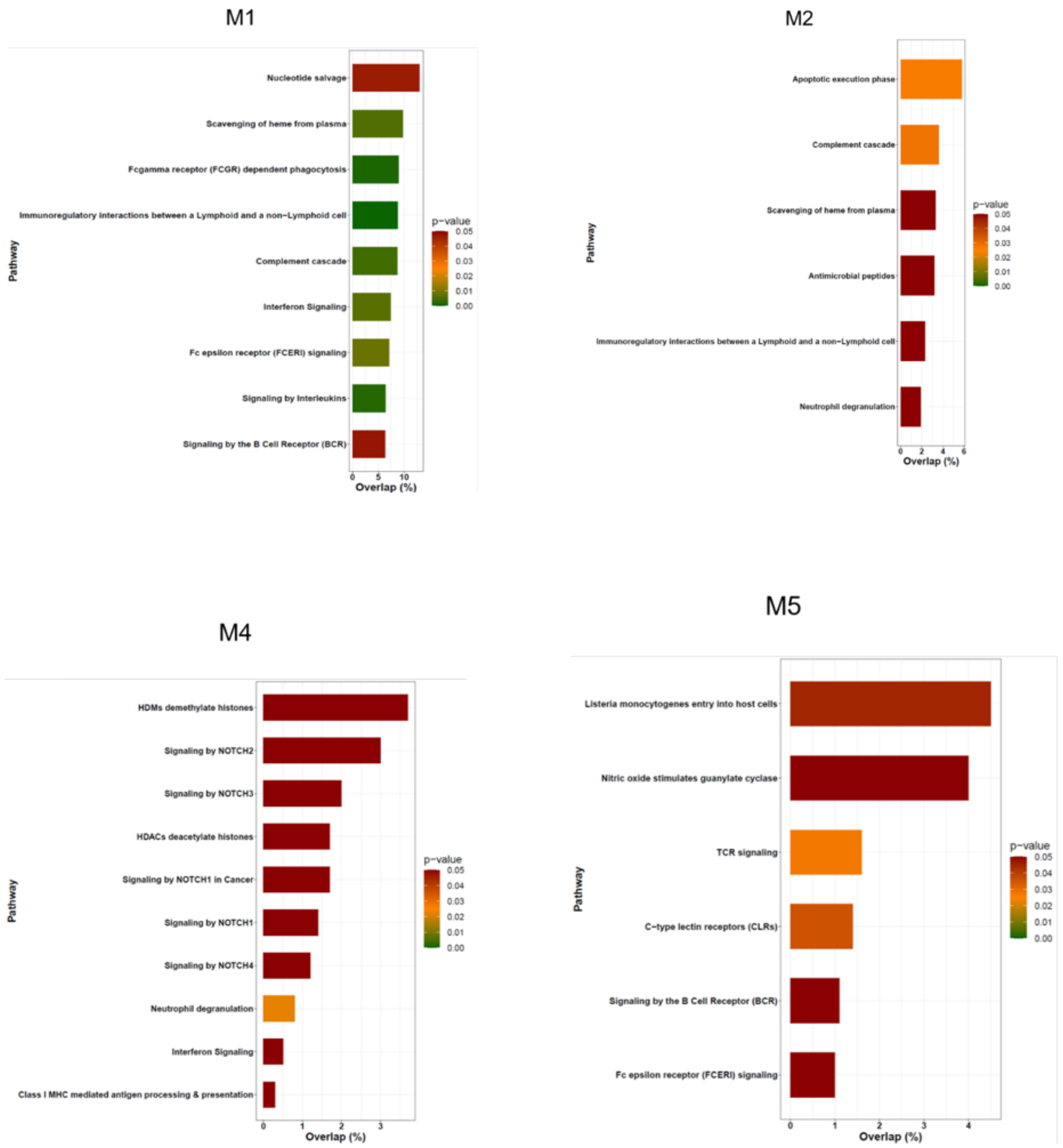

Figure S7. Functional enrichment analysis for co-expression modules M1 (A), M2 (B), M4 (C), M5 (D). Size of bars represent the representative rate of each pathway. Color of bars indicate statistical significance of the functional enrichment estimation.

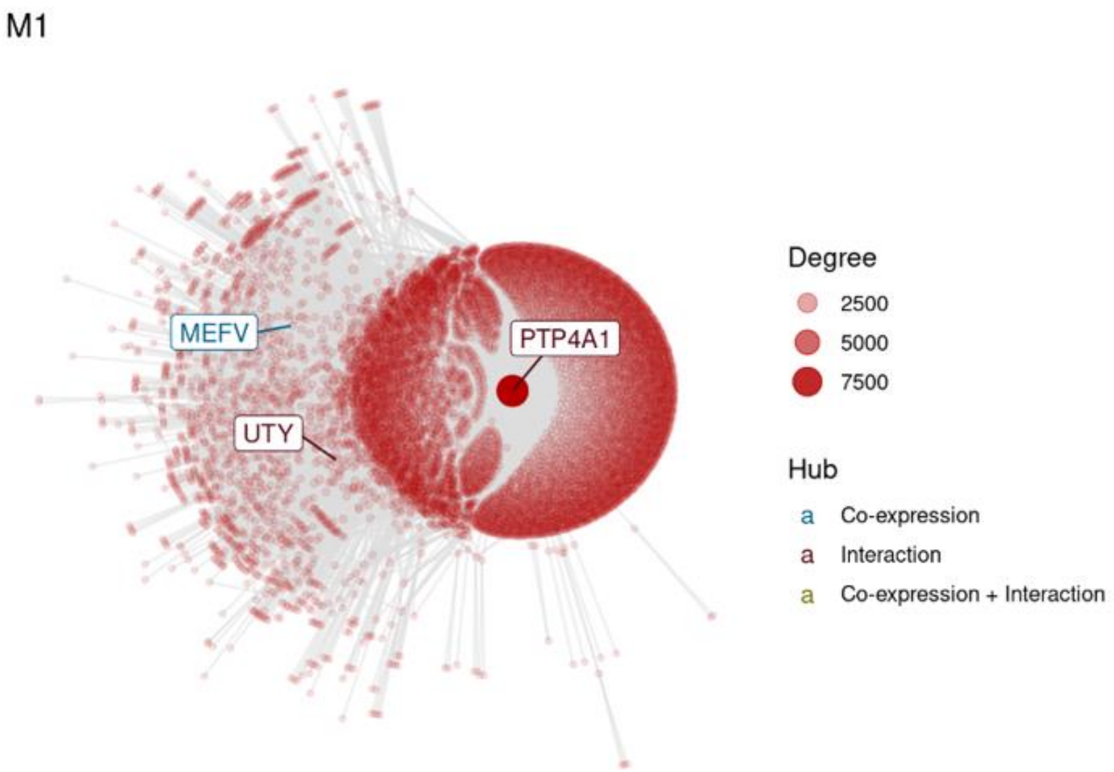

M2

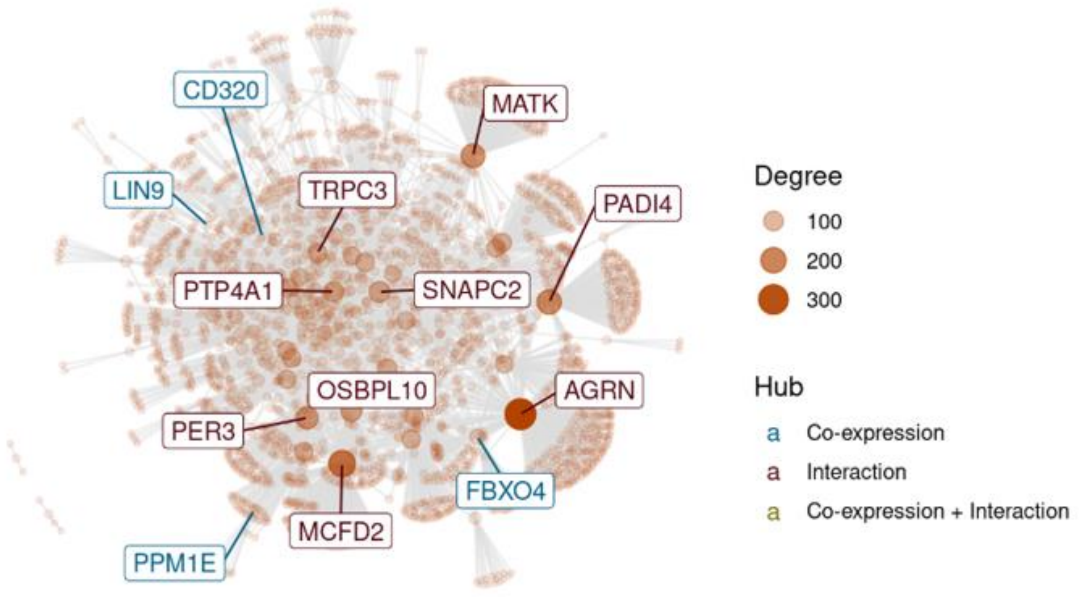
CD320
MATK
LIN9
TRPC3
PADI4
PTP4A1
SNAPC2
OSBPL10
AGRN
PER3
FBXO4
MCFD2
PPM1E
Degree
100
200
300
Hub
a Co-expression
a Interaction
a Co-expression + Interaction

M3

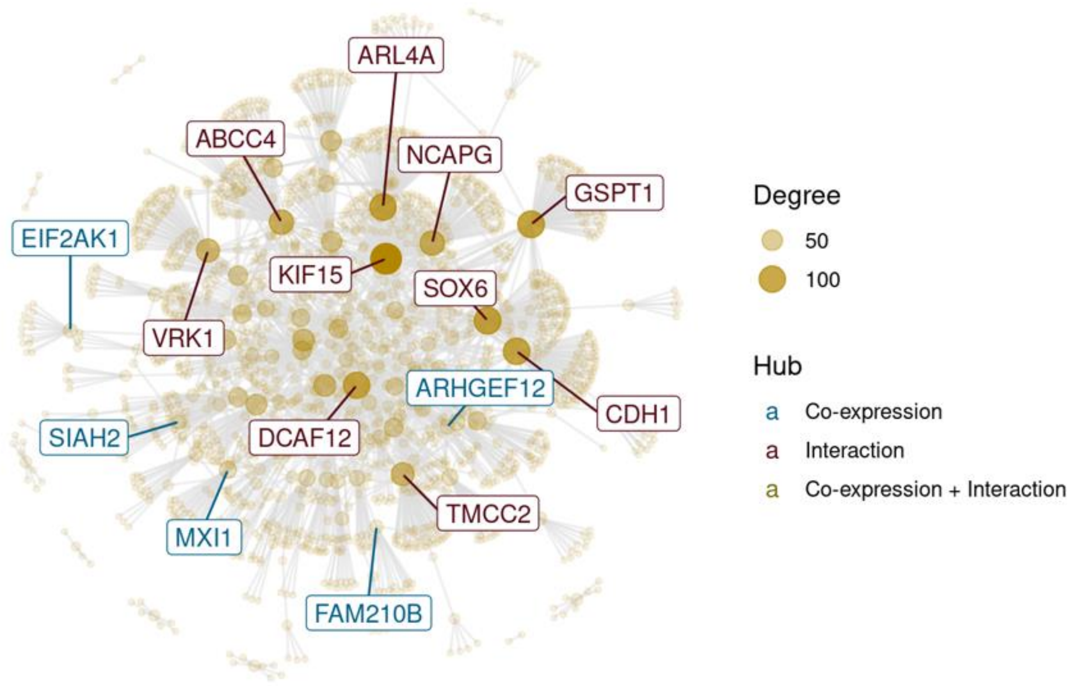
ARL4A
ABCC4
NCAPG
GSPT1
EIF2AK1
KIF15
SOX6
VRK1
ARHGEF12
CDH1
SIAH2
DCAF12
TMCC2
MXI1
FAM210B
Degree
50
100
Hub
a Co-expression
a Interaction
a Co-expression + Interaction

M4

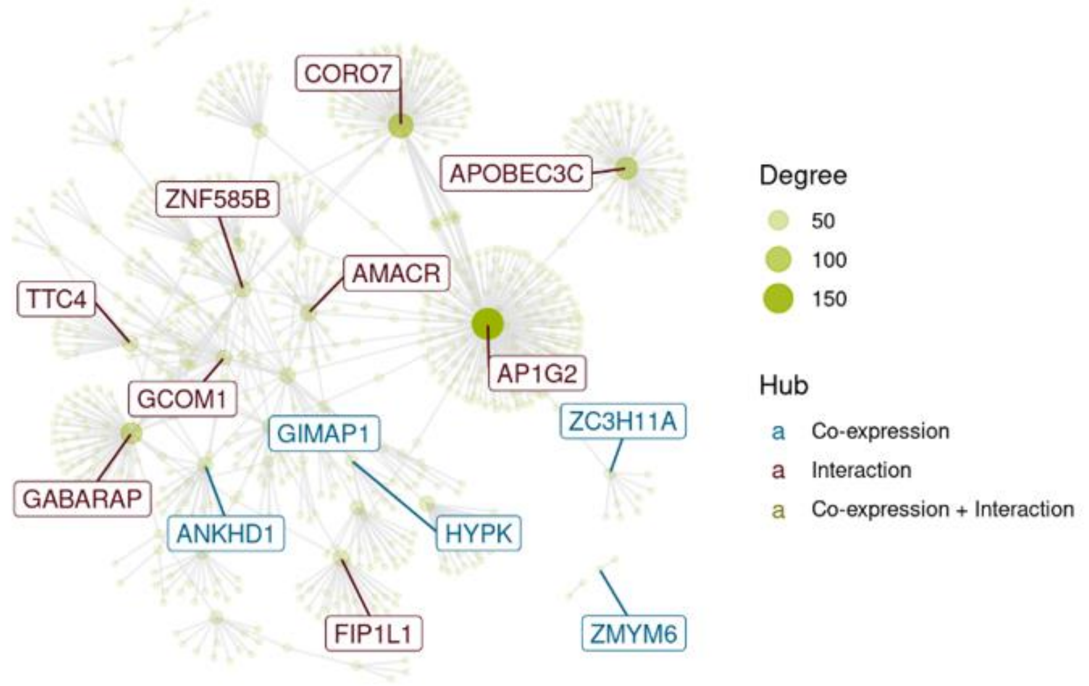
CORO7
APOBEC3C
ZNF585B
AMACR
TTC4
AP1G2
GCOM1
ZC3H11A
GIMAP1
GABARAP
ANKHD1
HYPK
FIP1L1
ZMYM6
Degree
50
100
150
Hub
a Co-expression
a Interaction
a Co-expression + Interaction

M5

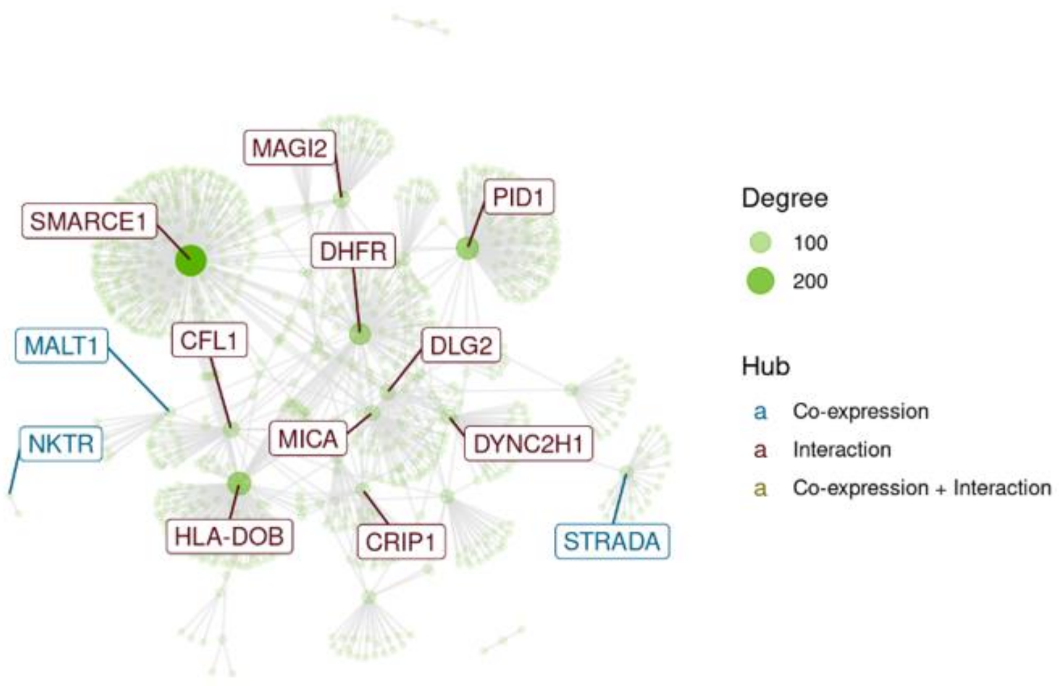

Figure S8. Interaction network between the genes comprising the co-expression modules M1, M2, M3, M4 and M5. There was no coincidence between the co-expression hub genes and the interaction hub genes in each module.
