# Supplementary material for: Molecular Insights into Cell-Mediated Immunity in Atypical Non-Ulcerated Cutaneous Leishmaniasis
Source: Microorganisms. 2025 Feb 13;13(2):413. doi: 10.3390/microorganisms13020413 (PMC11858551; doi:10.3390/microorganisms13020413)
Supplement: Supplementary file 1 [file microorganisms-13-00413-s001.zip › SUPPLEMENTARY_TABLES_MANUSCRIPT_RNA-seq_NUCL_FINAL_02-12-2024.pdf]

Table S1. Clinical outcome, origin – location of residence, number of lesions, term since symptoms manifestation, result in serological diagnoses (ELISA) for Immunoglobulin G (IgG) and IgM and size of lesion in Delayed-type hypersensitivity (DTH) of participants.

ID	Clinical outcome	Origin	N of lesions	Term of symptoms	IgG	IgM	DTH (mm)
1	NUCL	Amapala, Honduras	1	24 months	POS	POS	9
2	NUCL	Amapala, Honduras	20	12 months	POS	POS	12
3	NUCL	Amapala, Honduras	1	4 months	NEG	NEG	9
4	NUCL	Amapala, Honduras	1	-	POS	POS	0
5	NUCL	Amapala, Honduras	2	3 months	POS	POS	0
6	NUCL	Amapala, Honduras	5	12 months	POS	NEG	10
7	NUCL	Amapala, Honduras	1	2 months	NEG	NEG	0
8	NUCL	Amapala, Honduras	2	8 months	POS	POS	7
9	NUCL	Amapala, Honduras	1	36 months	POS	POS	17
10	NUCL	Amapala, Honduras	1	8 months	NEG	POS	6
11	VL	Amapala, Honduras	-	4 months	POS	POS	0
12	VL	Amapala, Honduras	-	15 days	POS	POS	0
13	AS	Amapala, Honduras	-	-	NEG	POS	11
14	AS	Amapala, Honduras	-	-	NEG	NEG	16
15	AS	Amapala, Honduras	-	-	POS	NEG	15
16	AS	Amapala, Honduras	-	-	POS	POS	11
17	AS	Amapala, Honduras	-	-	POS	NEG	14
18	AS	Amapala, Honduras	-	-	POS	POS	7
19	AS	Amapala, Honduras	-	-	POS	POS	16
20	AS	Amapala, Honduras	-	-	NEG	NEG	20
21	AS	Amapala, Honduras	-	-	NEG	POS	5
22	NEG	São Paulo, Brazil	-	-	NEG	NEG	-
23	NEG	São Paulo, Brazil	-	-	NEG	NEG	-
24	NEG	São Paulo, Brazil	-	-	NEG	NEG	-
25	NEG	São Paulo, Brazil	-	-	NEG	NEG	-
26	NEG	São Paulo, Brazil	-	-	NEG	NEG	-
27	NEG	São Paulo, Brazil	-	-	NEG	NEG	-
28	NEG	São Paulo, Brazil	-	-	NEG	NEG	-

NUCL – non-ulcerated cutaneous leishmaniasis, VL – visceral leishmaniasis, AS – asymptomatic, NEG -non-infected

Table S2. Statistically different differential expression between genes coding for biomarkers of Th1, Th2, Th17, CTL, Treg immune responses profiles, T lymphocyte exhaustion and innate immune response. The groups were compared two by two with parametric test-T student and non-parametric Mann-Witney (a), (b), (c): $p < 0.05$.

	**NULC vs. VL (a)**	**NULC vs. NEG (b)**	**VL vs. NEG (c)**	**NULC vs. VL and NULC vs. NEG (a,b)**	**NULC vs. NEG and VL vs. NEG (b,c)**	**NULC vs. VL and VL vs. NEG (a,c)**	**NULC vs. VL vs. NEG (a,b,c)**	**Sum**
**Th1**	1	2	1	1	6	3	15	29
**CTL**	0	1	2	1	1	0	6	11
**Th17**	1	0	0	1	4	3	10	19
**Treg**	0	0	1	0	3	4	10	18
**Th2**	1	0	0	0	1	8	15	25
**Exhaustion**	0	0	0	0	6	6	8	20
**Innate Immunity**	0	0	0	1	0	18	30	49
**Sum**	3	3	4	4	21	42	94	171

NUCL – non-ulcerated cutaneous leishmaniasis, VL – visceral leishmaniasis, AS – asymptomatic, NEG -non-infected

Table S3. Up-regulated differently expressed genes (DEGs) related to cell-mediated immunity (CMI) exclusive in NUCL or in VL and DEGs commonly expressed in NUCL and VL were selected from enriched pathways functionally related to CMI for the contrasts NUCL vs. NEG and VL vs. NEG. CMI Up-regulated commonly expressed DEGs were sorted in two groups: (i) more expressed in NUCL than in VL (NUCL > VL), (ii) more expressed in VL than NUCL. The Log fold-change (logFC) of each DEG and differences between VL and NUCL logFCs are presented.

NUCL		NUCL > VL				VL > NUCL				VL	
DEG	logFC	DEG	NUCL logFC	VL logFC	VL logFC - NUCL logFC	DEG	NUCL logFC	VL logFC	VL logFC - NUCL logFC	DEG	logFC
PDCD1	6,590	HLA-DQB1	2,759	1,439	-1,320	CDCA7	1,118	3,224	2,106	PSME4	2,603
LAG3	6,532	NCR1	1,518	1,274	-0,244	CRTAM	1,673	2,484	0,811	PSMA4	1,956
CD1A	5,333	KLRC1	2,436	2,217	-0,219	PDCD1LG2	1,281	1,760	0,479	PSMF1	1,737
VCAM1	5,269	CD274	0,992	0,943	-0,049	CD8A	1,048	1,530	0,482	PSMB1	1,647
CXADR	5,011	KLRD1	1,378	1,352	-0,026	HLA-DQB2	0,764	1,263	0,499	PSMC2	1,594
ICAM5	3,570	-	-	-	-	HLA-DRA	0,824	1,474	0,650	PSMD14	1,455
COLEC12	3,369	-	-	-	-	CD8B	0,768	1,438	0,670	PSMC6	1,449
-	-	-	-	-	-	HLA-DPA1	0,818	1,591	0,773	PSMA5	1,266
-	-	-	-	-	-	CD3D	0,803	1,929	1,126	PSMA7	1,214
-	-	-	-	-	-	-	-	-	-	PSMA1	1,100

Table S4. Down-regulated differently expressed genes (DEGs) related to cell-mediated immunity (CMI) exclusive in NUCL or in VL and DEGs commonly expressed in NUCL and VL were selected from enriched pathways functionally related to CMI for the contrasts NUCL vs. NEG and VL vs. NEG. CMI down-regulated commonly expressed DEGs were sorted in two groups: (i) more down-regulated in NUCL than in VL (NUCL > VL), (ii) more down-regulated in VL than NUCL. The Log fold-change (logFC) of each DEG and differences between VL and NUCL logFCs are presented.

NUCL		NUCL > VL				VL > NUCL				VL	
**DEG**	**logFC**	**DEG**	**NUCL logFC**	**VL logFC**	**VL logFC - NUCL logFC**	**DEG**	**NUCL logFC**	**VL logFC**	**VL logFC - NUCL logFC**	**DEG**	**logFC**
LPO	-2,607	ATP6V1E1	-0,650	0,000	0,650	SERPINA10	-0,681	-6,460	-5,778	TNFSF11	-5,369
MPO	-2,082	LTF	-2,577	-2,418	0,159	TREM1	-1,287	-5,558	-4,271	IL2RA	-4,049
ATP6V0E1	-0,617	-	-	-	-	CXCR2	-0,623	-4,822	-4,199	TNFSF14	-3,709
-	-	-	-	-	-	TNFRSF10C	-1,153	-4,928	-3,775	IL5RA	-3,082
-	-	-	-	-	-	CSF3R	-0,849	-3,716	-2,867	CXCL1	-3,033
-	-	-	-	-	-	FCER1A	-0,855	-3,700	-2,844	IL1R1	-2,945
-	-	-	-	-	-	ADGRG3	-1,073	-3,838	-2,764	GAB2	-2,944
-	-	-	-	-	-	FCGRT	-1,321	-4,066	-2,745	PIK3CD	-2,681
-	-	-	-	-	-	IL1R2	-0,842	-3,516	-2,674	TNFRSF1A	-2,616
-	-	-	-	-	-	CEACAM3	-1,332	-3,900	-2,568	TYK2	-2,434
-	-	-	-	-	-	NLRP12	-1,049	-3,403	-2,354	STAT5B	-2,256
-	-	-	-	-	-	CD163L1	-1,380	-3,715	-2,334	LYN	-2,178
-	-	-	-	-	-	PTAFR	-1,231	-3,521	-2,290	CSF1	-2,161
-	-	-	-	-	-	TBXAS1	-0,825	-3,073	-2,248	STAT3	-2,125
-	-	-	-	-	-	TNFAIP2	-0,579	-2,776	-2,197	TNFRSF8	-2,056
-	-	-	-	-	-	IGF2R	-1,020	-3,203	-2,184	TNFRSF13B	-1,547
-	-	-	-	-	-	NCF4	-1,200	-3,380	-2,179	IL10RB	-1,525
-	-	-	-	-	-	C5AR1	-1,118	-3,172	-2,054	ICAM1	-1,505
-	-	-	-	-	-	ITGAX	-0,887	-2,914	-2,027	JAK1	-1,325

-	-	-	-	-	-	SLC15A3	-0,875	-2,839	-1,964	CD27	-1,178
-	-	-	-	-	-	CSF2RA	-0,864	-2,809	-1,945	TNFRSF25	-1,122
-	-	-	-	-	-	FPR1	-1,143	-3,033	-1,890	-	-
-	-	-	-	-	-	FCGR3B	-1,876	-3,764	-1,888	-	-
-	-	-	-	-	-	HCK	-1,195	-2,992	-1,797	-	-
-	-	-	-	-	-	SLC11A1	-1,439	-3,211	-1,772	-	-
-	-	-	-	-	-	ALOX5	-0,955	-2,699	-1,744	-	-
-	-	-	-	-	-	CD14	-1,238	-2,970	-1,733	-	-
-	-	-	-	-	-	PILRA	-1,343	-3,074	-1,731	-	-
-	-	-	-	-	-	IL6R	-1,261	-2,989	-1,727	-	-
-	-	-	-	-	-	ORM1	-1,099	-2,792	-1,693	-	-
